# Deep learning in the stock market—a systematic survey of practice, backtesting, and applications

**DOI:** 10.1007/s10462-022-10226-0

**Published:** 2022-06-30

**Authors:** Kenniy Olorunnimbe, Herna Viktor

**Affiliations:** School of Electrical Engineering and Computer Science, University of Ottawa, Ottawa, ON Canada

**Keywords:** Deep learning, Machine learning, Neural network, Stock market, Financial market, Quantitative analysis, Backtesting, Practice and application

## Abstract

The widespread usage of machine learning in different mainstream contexts has made deep learning the technique of choice in various domains, including finance. This systematic survey explores various scenarios employing deep learning in financial markets, especially the stock market. A key requirement for our methodology is its focus on research papers involving backtesting. That is, we consider whether the experimentation mode is sufficient for market practitioners to consider the work in a real-world use case. Works meeting this requirement are distributed across seven distinct specializations. Most studies focus on trade strategy, price prediction, and portfolio management, with a limited number considering market simulation, stock selection, hedging strategy, and risk management. We also recognize that domain-specific metrics such as “returns” and “volatility” appear most important for accurately representing model performance across specializations. Our study demonstrates that, although there have been some improvements in reproducibility, substantial work remains to be done regarding model explainability. Accordingly, we suggest several future directions, such as improving trust by creating reproducible, explainable, and accountable models and emphasizing prediction of longer-term horizons—potentially via the utilization of supplementary data—which continues to represent a significant unresolved challenge.

## Introduction

Technology has long substantially enabled financial innovation (Seese et al. 2008). In Insights (2019), Deloitte surveyed over 200 US financial services executives to determine their use of Artificial Intelligence (AI) and its impact on their business. A total of 70% of respondents indicated that they use general-purpose Machine Learning (ML), with 52% indicating that they use Deep Learning (DL). For these respondents, the most common uses of DL are reading claims documents for triage, providing data analytics to users through intuitive dashboards, and developing innovative trading and investment strategies.

The Institute for Ethical AI & Machine Learning (EAIML) has developed eight principles for responsible ML development; these include pertinent topics such as explainability, reproducibility, and practical accuracy (The Institute for Ethical AI & Machine Learning 2020). Recent research has emphasized the issue of Explainable AI (XAI) and Reproducible AI (Gundersen et al. 2018) in numerous application domains. In a survey on XAI, the need for interpretable AI was identified as a major step toward artificial general intelligence (Adadi and Berrada 2018). However, more work is needed to ensure domain-specific metrics and considerations are used to assess applicability and usability across diverse ML domains.

Paleyes et al. (2020) suggest *practical consideration* in deploying ML for production use: “The ability to interpret the output of a model into understandable business domain terms often plays a critical role in model selection, and can even outweigh performance consideration.” For example, Nascita et al. (2021) fully embraces XAI paradigms of trustworthiness and interpretability to classify data generated by mobile devices using DL approaches.

In the domain of financial analysis using stock market data, a key tool for achieving explainability and giving research a good chance at real-world adoption is *backtesting* (de Prado 2018; Arnott et al. 2018). This refers to using historical data to retrospectively assess a model’s viability and instill the confidence to employ it moving forward. This is based on the intuitive notion that any strategy that worked well in the past is likely to work well in the future, and vice versa (de Prado 2018).

Numerous surveys have considered applications of DL to financial markets (Jiang 2021; Zhang et al. 2021; Hu et al. 2021; Li and Bastos 2020; Ozbayoglu et al. 2020), with (Ozbayoglu et al. 2020) considering numerous financial applications to demonstrate that applications involving stock market data, such as algorithmic trading and portfolio management, present the most interesting cases for researchers. Elsewhere, (Jiang 2021) focuses on DL research in the stock market, especially research concerning reproducibility; however, despite presenting financial metrics, there is no indication of backtesting or practicality. Meanwhile, (Hu et al. 2021) presents an analysis based on evaluation results such as bins of accuracy results and ranges of returns that, nonetheless, offers no clear explanation for different kinds of metrics and does not consider XAI.

The authors of Li and Bastos (2020) emphasize the importance of evaluations using financial metrics but limit their focus to profitability as a financial evaluation. Although they do discuss volatility, this is not considered for evaluation because it can result in poor financial returns despite its high level of accuracy. This survey explores the strategies that various researchers have employed to understand DL in the stock market, focusing on studies addressing explainability, reproducibility, and practicality. To the best of our knowledge, this work represents the first study to adopt backtesting and domain-specific evaluation metrics as primary criteria. This is represented by the following specific questions:

### Question 1

What current research methods based on deep learning are used in the stock market context?

### Question 2

Are the research methods consistent with real-world applications, i.e., have they been backtested?

### Question 3

Is this research easily reproducible?

To answer question 2, we focus on works that were backtested as part of the research methodology. Proper backtesting provides assurance that the algorithm has been tested in different time horizons, consistent with domain-specific considerations, which improves investor confidence and makes its application in a real-world trading scenario more likely  (Arnott et al. 2018). This serves as the primary criteria for the literature reviewed. For question 3, we consider not only works where the source data and code are provided but also on works the research could be reproduced. Section 4 further explains the approach employed and the search criteria.

Section 2 explains the characteristics, types, and representations of stock market data. Then, Sect. 3 discusses applications of DL in the stock market. We begin the section by summarizing the different DL techniques currently used in the stock market context and conclude by itemizing the specific ways these techniques are applied to stock market data. In Sect. 4, we elaborate on our research questions, answering the research questions by summarizing our survey findings. Section 5 presents challenges remaining to be unresolved and future research directions, and Sect. 6 concludes the survey.

## Understanding stock market data

Not unlike other ML applications, data represents a crucial component of the stock market learning process (de Prado 2018). Understanding the different forms of data that are employed to utilize DL for the stock market substantially contributes to enabling proper identification of our data requirements in accordance with the task in question. This section considers the different characteristics, types, and representations of data that are relevant to mining stock market data using DL. Notably, as will become evident, some of these data forms are quite specific to stock market data.

### Data characteristics

#### Source

Although trading venues such as stock exchanges are often perceived as the main source of stock market data, in recent years, other data sources, including news articles and social media, have been explored as data sources for ML processes (Day and Lee 2016; Haibe-Kains et al. 2020; Yang et al. 2018; Adosoglou et al. 2020). There is a direct correlation between data source and data type, as Sect. 2.2 demonstrates. Data source also largely depends on the intended type of analytics. If the goal is a simple regression task using purely historical market data, then the primary or only source could be trading data from the trading venue. For more complicated tasks, such as studying the effect of user sentiments on stock movement, it is common to combine trading data with data obtained from social media services or comments on relevant news articles. Irrespective of complications associated with the task at hand, it is rare to not use the trading venue as a source because literal data is always integral. Although several of the studies considered do not incorporate trading data—e.g., (Bao and Liu 2019; Ferguson and Green 2018)—these are generally theoretical studies that utilize simulated data.

#### Frequency

Data frequency concerns the number of data points within a specific unit of time (de Prado 2018). What any particular data point captures can be reported in different ways, from being represented as an aggregate (e.g., min, max, average) to using actual values. Data granularity can range from a daily snapshot (typically the closing value for trading data) to a fraction of a second for high-frequency market data. A more established representation of stock market data as *bars* (Sect. 2.3.1) refers to presenting multiple data points as an understandable aggregate of the highlights within that time interval.

For non-traditional data sources, such as news or social media, it is quite common to combine and summarize multiple individual items within the same time interval. For example, (Day and Lee 2016) uses multiple daily news headlines as part of the training data. Elsewhere, using a sentence encoder (Conneau et al. 2017) generates equal length vectors from differently sized sets of words representing different sentences. The literature reviewed commonly uses a snapshot or aggregated data to summarize a data point within a time interval. This could be due to the data’s granularity being directly proportional to its volume. Consequently, more parameters will be required in neural networks comprising highly granular data.

#### Volume

Although the volume of the data closely relates to the frequency of the data and the specific unit of data (de Prado 2018), we should differentiate volume from frequency because, while a high frequency typically translates to a relatively high volume, volume size might not directly correlate to data frequency. This becomes more apparent when we consider seasonality or holidays for the same time interval. We can also recognize that, based on the time of day, the volume of data generated for the same subject of interest within the same period could be vastly different, suggesting a differential occurrence rate. This is particularly relevant for non-conventional data types, such as news and social media data, where high volume (i.e., the size of the volume) might not be directly correlated to data frequency. This becomes more apparent when we consider seasonality or holidays for the same time interval. We can also notice that based on the time of day, the volume of data generated for the same subject of interest within the same period could be vastly different, suggesting a different rate of occurrence. This is particularly relevant for non-conventional data types, such as news or social media data.

Using Apple Inc. as an example (Investing.com 2013), a day marking a product announcement produces a substantially larger volume of news articles and relevant social media content than other days. Although this content might not affect the volume of the trading data—which depends more heavily on market data frequency—such instances might produce noticeable differences in the rate of change in market values. An increased rate warrants a different level of attention compared to a typical market day. The relationship between market data frequency and alternative data volume itself represents an interesting area of research that deserves a special level of attention.

Understanding data volume and data frequency is critical to designing infrastructure for processing data. As data volume approaches the realm of big data, precluding efficient computation in memory, it is necessary to consider alternative ways of processing data while utilizing relevant components of that data. Here, we begin considering ways of parallelizing the learning process without losing relationships between parallel batches. Data processing at such a scale requires parallel processing tools, such as those described by Zaharia et al. (2010).

### Data types

#### Market data

Market data are trading activities data generated by trading venues such as stock exchanges and investment firms. They are typically provided via streaming data feeds or Application Programming Interface (API) used within protocols such as the Financial Information eXchange (FIX) and the GPRS Tunnelling Protocol (GTP) (Wikipedia 2020d) (accessed 19-Aug-2020). A typical trade message concerning stock market data comprises a ticker symbol (representing a particular company), bid price, ask price, time of last quote, and size of the sale (Table 1).

**Table 1:** A sample trade message for Apple Inc. (AAPL)

Ticker symbol	AAPL
Name	Apple Inc.
Last trade price	289.80
Last trade timestamp	1577480401
Last trade volume	35447203
Exchange	NASDAQ

For messages with quote data, we should expect to see both the *bid* price & volume and the *ask* price & volume. These represent how much people are willing to buy and sell the asset at a given volume. Market data represent the core data type used by ML research in the stock market context and typically provide a detailed representation of trading activities regarding market assets such as equities/shares, currencies, derivatives, commodities, and digital assets. Derivatives can be further broken down into futures, forwards, options, and swaps (Derivative 2020).

Market data can be either real-time or historical (de Prado 2018). Real-time data are used to make real-time trading decisions about buying and selling market instruments. Historical data are used to analyze historical trends and make informed decisions regarding future investments. Typically, historical data can contain intraday or end-of-day data summaries. The granularity of real-time data can be as detailed as a fraction of a second, with some tolerance for short delays. Comparing data for the same period, the frequency of a real-time data feed is expected to be much higher than historical data.

We can further separate market data, based on the details it contains, into Level I and Level II market data. Level II data contains more information and provides detailed information on bids and offers at prices other than the highest price (Zhang et al. 2019). Level I data generally contain the basic trading data discussed thus far. Level II data are also referred to as order book or depth of book because they show details of orders that have been placed but not yet filled. These data also show the number of contracts available at different bid and ask prices.

#### Fundamental data

Unlike market data, where data directly relate to trading activity on the asset of interest, fundamental data are based on information about the company the asset is attached to Christina Majaski (2020). Such data depict the company’s standing using information such as cash flow, assets, liabilities, profit history, and growth projections. These kinds of information can be obtained from corporate documentation such as regulatory filings and quarterly reports. Care has to be taken to confirm whether fundamental data points are publicly available because these are typically reported with a lapse. This means that analyzing the data must align properly with the date it became publicly available and not necessarily the date the report was filed or indexed.

Notably, some fundamental data are reported with some data yet to be made available, becoming backfilled upon availability. When fundamental data are published before source data becomes available, placeholder values are used during the interim period. Furthermore, given companies can issue revisions or corrections to sources multiple times, these will need to be corrected in the fundamental data, which suggests the need to incorporate a backfilling technique into the data consumption design. By definition, the frequency of this kind of data is very low compared to market data. This might explain why limited DL literature employs fundamental data. However, this also indicates the existence of a gap in research utilizing this kind of data, which would ideally be filled by considering fundamental data alongside other data types to provide a significant learning signal that remains to be fully exploited.

#### Alternative data

Alternative data represents any other unconventional data type that can add value to already-established sources and types (de Prado 2018). This can range from user-generated data (e.g., social media posts, financial news, and comments) to Internet-of-Things data (e.g., data from different sensors and devices). Alternative data typically complement the aforementioned data types, especially market data. Given the nature of alternative data, they are typically much larger, hence requiring a sophisticated processing technique.

Notably, alternative data includes a vast amount of data that is open to interpretation because the signal might not be immediately obvious. For example, a market participant interested in Apple Inc. stocks might choose to observe different news articles related to the company. Although there might be no direct reports about the company releasing a new product line, news reports about key meetings or large component purchases can indicate the plausibility of action. Accordingly, stock market professionals and researchers have become attentive to such indirect signals, and now consider alternative data essential to their data pipeline. Numerous researchers now combine traditional data types with either or both news article and social media content to make market predictions. Social media especially has become a very popular alternative data type, primarily due to its position in the mainstream.

Table 3 presents certain representative attributes of the different data types. All of the attributes associated with market data and fundamental data are numerical and aggregated based on the available time series. For example, the intraday market data entry in row 1 of Table 2 shows the open and close prices for a one-hour time window that begins at 10 am and ends at 10:59 am. It also includes the maximum and minimum price and the total volume traded within the same window (Table 3).

**Table 2:** Intraday time bar for ticker IBM

Date	Time	Open	High	Low	Close	Volume
20160128	10:00	122.17	122.27	122.09	122.09	4,934
20160128	11:00	121.42	121.60	121.38	121.52	12,254

**Table 3:** Representative attributes by data types

Market data attributes	Fundamental data attributes	Alternative data attributes
open price, high price, low price, close price, volume	revenue, earnings per share, market capitalization, dividend, average volume, shares outstanding, next earning date	google trends, news, texts, tweets, satellite imagery

A fourth data type known as *Analytics data* (de Prado 2018), describes data derived from any of the other three types. Attributes of analytics data are earnings projections or sentiments from news or tweets that are combined with trade volume. We have chosen not to include this category because it does not clearly represent a direct source, and it is usually unclear what heuristics have been used to obtain the derived data points. Furthermore, given the objective of academic research is to make the metrics explicit, it is counter-intuitive to consider them useable input.

Table 4 presents the characteristics of the data employed by the literature reviewed, including the aforementioned data types. It is apparent that market data represents the most common type, with actual trading prices and volumes often paired with fundamental data to compute technical indicators (Soleymani and Paquet 2020; Wang et al. 2019b). Table 5 presents a more complete representation of freely or publicly available data sources that fully itemizes attributes.

**Table 4:** Characteristics of data in survey

Source	Type	Frequency	Free	Library
https://www.investing.com	Market, fundamental	Interday	Y	Investpy
https://www.wrds-www.wharton.upenn.edu^a^	Market, fundamental	Interday, intraday	N	Na
https://www.bloomberg.com	Market, fundamental	Interday, intraday	N	Na
https://finance.yahoo.com	Market, fundamental	Interday, intraday	Y	yfinance
https://www.kaggle.com	Market	Interday*	Y	Kaggle-api
https://www.interactivebrokers.com	Market, fundamental	Interday, intraday	N	Tws-api
https://www.taifex.com.tw	Taiwan market	Interday, intraday	Y	Na
pypi.org/project/tushare	China market, fundamental	Interday, intraday	Y	Tushare
https://optionmetrics.com	Market, fundamental	Interday, intraday	N	Na
https://www.refinitiv.com	Market, fundamental	Interday, intraday	N	Na
https://datashop.deutsche-boerse.com	Market, fundamental	Interday, intraday	N	Na
https://www.trkd.thomsonreuters.com	Market, fundamental	Interday, intraday	N	Na
https://www.wind.com.cn	China market, fundamental	Interday, intraday	N	Na
https://etsin.fairdata.fi	Nordic market	Intraday*	Y	Na
https://www.londonstockexchange.com	UK market	Interday	N	Na
https://pinnacledata2.com	Narket, fundamental	Interday, intraday	N	Na
http://www.apex.com.tw	Taiwan market, fundamental	Interday, intraday	N	Na
https://www.joinquant.com	China market, fundamental	Interday, intraday	N	Jqdatasdk

*Subject to availability

^a^WRDS—compustat daily updates

**Table 5:** Characteristics of public data sources

Source	Market data attributes	Fundamental data attributes	Frequency
investing^a^	Open price, high price, low price, close price, volume	Revenue, earnings per share, market capitalization, dividend, average volume, ratio, beta, shares outstanding, next earning date	Daily, weekly, monthly
y-finance^b^	Open price, high price, low price, close price, volume	Major holders, institutional holders, mutual fund holders, dividends, splits, actions, calendar, earnings, quarterly earnings, financials, quarterly financials, balance sheet, quarterly balance sheet, cashflow, quarterly cashflow, sustainability, shares outstanding	1 min, 2 min, 5 min, 15 min, 30 min, 60 min, 90 mins, 1 h, 1 day, 5 days, 1 week, 1 month, 3 months
taifex^c^	Open bid, high bid, low bid, last bid, volume, best bid, best ask, historical high, historical low	Not available	Daily
kaggle^d^	Open price, high price, low price, close price, volume	Not available	Daily
tushare^e^	Open price, high price, low price, close price, volume	Account receivable turn day, account receivable turnover, business income, current asset days, current asset turnover, earnings per share, earnings per share (year over year), fixed assets, gross profit rate, inventory days, inventory turnover, liquid assets, net profit ratio, net profits, outstanding, profits (year over year), report date, reserved, reserved per share, return on equity, time to market, total assets	Daily
etsin^f^	Open price, high price, low price, close price, volume	Not available	Daily

^a^https://github.com/alvarobartt/investpy

^b^https://github.com/ranaroussi/yfinance

^c^https://www.taifex.com.tw/enl/eng3/totalTableDate

^d^https://www.kaggle.com/datasets, https://github.com/kaggle/kaggle-api

^e^https://github.com/waditu/tushare

^f^https://etsin.fairdata.fi/

Sources including investing.com, finance.yahoo.com and kaggle.com utilize either API or libraries, facilitating interactions with them and unlocking better integration with the ML system. Sources without any programmatic interface usually make data available as manual downloadable files.

The other major factor that affects the preferred data source is the frequency of availability, for example, whether the data is available multiple times a day (intraday data) or once a day (interday data). Given the potential volume and size of historical data, it is common for intraday data to remain available for a shorter timeframe than interday data, especially for freely available data sets. However, in most cases, it is possible to pay for intraday data for a longer timeframe if required for lower latency projects.

### Data representation

Data generated from the stock market are typically represented as *Bars* and *Charts*. It is worth discussing these representations because they represent the most typical forms of representing data either numerically (bars) or graphically (charts).

#### Bars

Bars enable extraction of valuable information from market data in a regularized manner (de Prado 2018). They categorize futures into standard and more advanced types, with the advanced types comprising derivative computation from standard types. However, standard types are more common and also form the basis of chart representation.

**Fig. 1:**
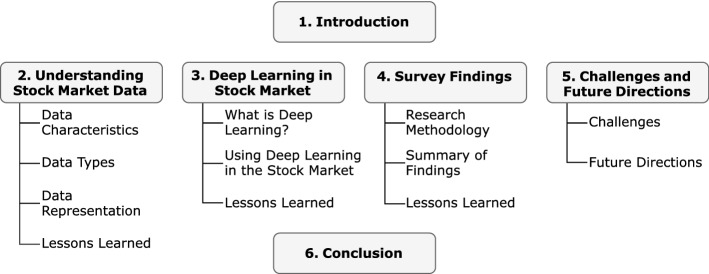
Survey structure

Standard bars help to summarize market data into equivalent intervals and can be used with both intraday and historical data (Fig. 1). The different types of standard bars all typically contain certain basic information for the specified interval, including the *timestamp, Volume-Weighted Average Price (VWAP), open price, close price, high price, low price*, and *traded volume*, all within the specified interval. The VWAP is based on the total traded for the day, irrespective of the time interval, and is computed as $\sum price \cdot volume/\sum volume$. The different standard bars are described in the following paragraphs.

**Fig. 2:**
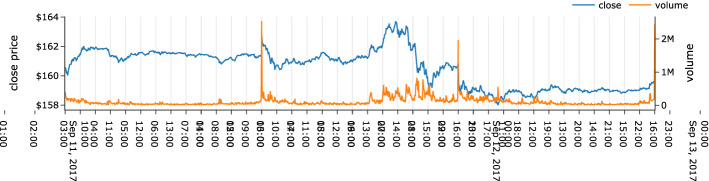
Intraday tick time series showing trade price and volume within the trading hours, across 2 days (Investing.com 2013)

*Time bars* This is the most common bar type and derives from summarizing data into an equivalent time interval that includes all of the aforementioned standard bar information. Intraday hourly time bars feature hourly standard bar information for every hour of the day. For historical data, it is common to obtain details for each day. Table 2 exemplifies intraday time bars that can capture information.

The VWAP assists by demonstrating the trend for the price of a traded item during a given day. This single-day indicator is reset at the start of each trading day and should not be used in the context of daily historical data.

*Tick bars* Unlike time bars that capture information at regular time intervals, tick bars capture the same information at a regular number of transactions or *ticks*. Ticks are trades in the stock market that can be used to represent the movement of price in trading data (i.e., the *uptick* and *downtick*). Ticks are commonly used for different stages of modeling market data, as in the case of *backtesting*. However, historical stock market data are not as freely accessible in the form of tick bars, especially for academic research purposes. For this purpose, most of the literature reviewed uses time bars, despite its statistical inferiority for predictive purposes.

*Volume bars* Although tick bars exhibit better statistical properties than time bars (i.e., they are closer to independent distribution), they still feature the shortcoming of uneven distribution and propensity for outliers (de Prado 2018). This can be because a large volume of trade is placed together from accumulated bids in the order book, which gets reported as a single tick, or because orders are equally recorded as a unit, irrespective of size. That is, an order for 10 shares of a security and an order for 10,000 shares are both recorded as a single tick. Volume bars help to mitigate this issue by capturing information at every predefined volume of securities. Although volume bars feature better statistical properties than tick bars (Easley et al. 2012), they are similarly seldom used in academic research.

*Range bars* Range bars involve information being captured when a predefined monetary range is traded. They are also referred to as dollar bars (de Prado 2018). Range bars are particularly useful because, by nature, securities appreciate or depreciate constantly over a given period. Consider a security that has depreciated by 50% over a certain period; by the end of that period, it is possible to purchase twice as much as at the beginning. For instance, consider a security that has depreciated from $100 to $50 over a given period. A capital investment of $1000 would only have obtained 10 units at the start of the depreciation period; however, at the end of the period, that investment can obtain 20 units. Furthermore, corporate actions (e.g., splits, reverse splits, and buy-backs) do not impact range bars to the extent that they impact ticks and volume bars.

#### Charts

Charts visually represent the aforementioned bars, especially time bars. It might not be clear how these are relevant to a survey of DL applications in the stock market context, given it is possible to use the actual data that the charts are based on. However, various novel applications have used charts as training data. For example, (Kusuma et al. 2019) uses the candlestick plot chart as the input image for a Convolutional Neural Network (CNN) algorithm. The charts most commonly used to visually represent stock market data are line, area, bar, and candlestick charts. Of interest here, however, are the candlestick and bar charts, which visually encode valuable information that can be used as input for DL algorithms.

**Fig. 3:**
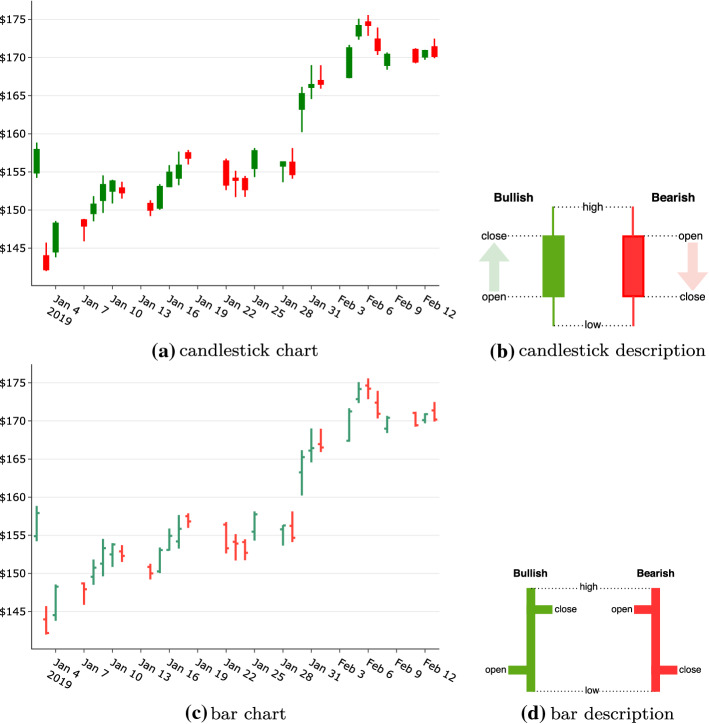
Candlestick & bar charts

Candlestick and bar charts can visually represent *Open-High-Low-Close (OHLC)* data, as Figure 3 shows. These two types of charts are optionally color-coded, with red indicating bearish (closing lower than it opened) and green indicating bullish (closing higher than it opened). By properly encoding this information into these charts, an algorithm such as CNN can interpret numerous signals to generate an intelligent model.

### Lessons learned

The distinctive structure and differential representations of stock market data cannot be overestimated. This section considers some of these differences, especially those used in stock-market implementations of ML algorithms using DL. Understanding data characteristics based on specific use cases can determine a given data set’s suitability for the intended use case. By understanding the different types of data used in the stock market, we can refer to the data types needed, which closely relate to their characteristics. For example, given the nature of alternative data, we can expect it to feature significant volume, especially in comparison to fundamental data.

The frequency of data also varies significantly by type. Understanding the granularity of the intended task enables determination of the frequency of the data to be obtained. For example, intraday market data will be required for modeling tasks requiring minute- or hour-level data. This also affects the volume of data required. It is interesting to note data representation, especially market data. The required frequency guides data representation as summarized time bars rather than tick-by-tick data.

Chart representations of market data also provide novel ways of learning from visual representations. Candlestick and bar charts convey information at a rich and detailed level worthy of exploitation as a learning source. Nonetheless, this is accompanied by the complex task of consuming the image rather than the data that it is based upon and, although  (Kusuma et al. 2019) used a candlestick chart for this purpose, the authors failed to compare the performance with the performance using the raw data. It would be interesting to observe comparisons of results for raw data and visual representations of that same data.

## Deep learning for stock market applications

### What is deep learning?

Deep learning describes an ML technique based on networks of simple concepts and featuring different arrangements or architecture that allows computers to learn complicated concepts from simple nodes that are graphically connected using multiple layers (Goodfellow et al. 2016). The resurgence of DL was led by probabilistic or Bayesian models such as Deep Belief Networks (DBN)  (Hu et al. 2021; Goodfellow et al. 2016), which comprise nodes representing random variables with probabilistic relationships to each other. More recently, however, Artificial Neural Networks (ANN) that comprise nodes representing neurons that are generated by the training process have witnessed increasing popularity. All of the architectures we encounter in this survey are based on ANN; this section details these architectures.

Generally speaking, ANN are information processing systems with designs based on the human nervous system, specifically the brain, and that emphasize problem-solving (Castro 2006). Typically, they comprise many simple processing elements with adaptive capabilities that can process a massive amount of information in tandem. Given neurons are the basic units for information processing in the brain, their simplified abstraction forms the foundation of ANN. The features and performance characteristics that ANN share with the human nervous system are (Castro 2006):

1. The initial information processing unit occurs in elements known as *neurons*, *nodes* or *units*.
2. Neurons can send and receive information from both each other and the environment.
3. Neurons can be connected, forming a connection of neurons that can be described as *neural networks*.
4. Information is transmitted between neurons via connection links called *synapses*.
5. The efficiency of synapses, represented by an associated *weight* value or *strength*, corresponds, in aggregate, to the information stored in the neural network.
6. To acquire knowledge, connective strengths (aggregated weight values) are adapted to the environmental stimuli, a process known as *learning*.

Patterns are created by the information stored between neurons, which represents their synaptic or connective strength (Goodfellow et al. 2016). Knowledge is represented to influence the course of processing, which becomes a part of the process itself. This invariably means that learning becomes a matter of finding the appropriate connective strength to produce satisfactory activation patterns. This generates the possibility that an information processing mechanism can learn by tuning its connective strength during the processing course. This representation also reveals that knowledge is distributed over the connections between numerous nodes, meaning no single unit is reserved for any particular pattern.

Thus, an ANN can be summarized according to these three key features:

1. A set of *artificial neurons*, also known as nodes, units, or neurons.
2. A method for determining weight values, known as *training* or *learning* techniques.
3. A pattern of connectivity, known as the *network architecture* or *structure*.

The following sections detail these three features.

#### Artificial neurons

A biological neuron primarily comprises a *nucleus* (or *soma*) in a *cell body* and *neurites* (*axons* and *dendrites*) (Wikipedia 2020b). The axons send output signals to other neurons, and the dendrites receive input signals from other neurons. The sending and receiving of signals take place at the *synapses*, where the sending (or *presynaptic*) neuron contacts the receiving (or *postsynaptic*) neuron. The synaptic junction can be at either the cell body or the dendrites. This means that the synapses are responsible for signal/information processing in the neuron, a feature that allows them to alter the state of a postsynaptic neuron, triggering an electric pulse (known as *action potential*) in that neuron. The spikes cause the release of neurotransmitters at the axon terminals, which form synapses with the dendrites of other neurons. The action potential only occurs when the neuron’s intrinsic electric potential (known as *membrane potential*) surpasses a threshold value.

**Fig. 4:**
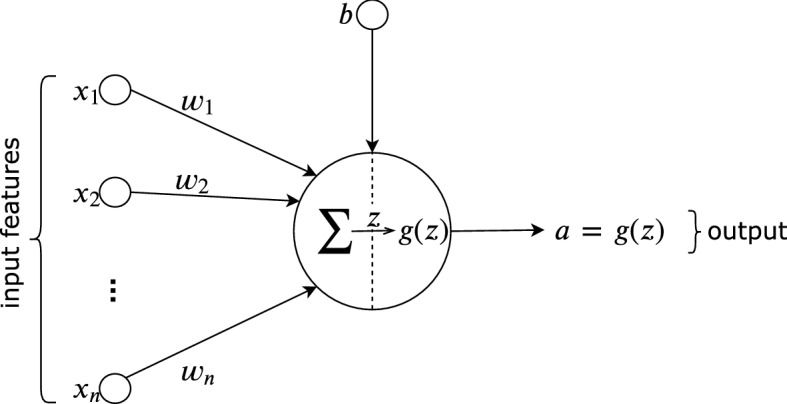
Model of a typical neuron (Castro 2006)

An *artificial neuron* attempts to emulate these biological processes. In an artificial neuron, the synapse that connects the input to the rest of the neuron is known as a *weight*, characterized by *synaptic strength, synaptic efficiency, connection strength*, or *weight value*. Figure 4 show a typical artificial neuron.

As each input connects to the neuron, it is individually multiplied by the synaptic weight at each of the connections, which are aggregated in the *summing junction*. The summing junction adds the product of all of the weighted inputs with the neuron’s bias value, i.e., $z = \sum \mathbf {wx}+ b$. The images essentially represent this. The *activation function* (also referred to as the *squashing function*) is represented as $g(z)$ and has the primary role of limiting the permissible value of the summation to some finite value. It determines a neuron’s output relative to its net input, representing the summing junction’s output. Thus, the neuron’s consequent output, also known as the *activation* ($a$), becomes:

1 $$\begin{aligned} a = g(z) = g\bigg ( \sum _{j=1}^n w_j{}x_j + b \bigg ) \end{aligned}$$

During the learning process, it is common to randomly initialize the weights and biases. These parameters are used by the activation to compute the neuron’s output. In this simple representation of one neuron, we can imagine that the output (prediction) of the neuron is compared with the input (true value) using a *loss function* to generate the error rate. Through an optimization method called *Stochastic Gradient Descent*, the error rate is propagated back to the network, a process called *backpropagation* (Rumelhart et al. 1986). This process is repeated over multiple iterations or *epochs* until a defined number of iterations is achieved or the error rate falls below a satisfactory threshold.

Multiple types of activation functions (Wikipedia 2020b) are used across different neural network architectures. The Rectified Linear Unit (ReLU) activation function has been more popular in recent applications of Feed-Forward Neural Networks (FFNN) because it is not susceptible to the vanishing gradient issue (Wikipedia 2020c), which impacts use of the sigmoid function across multiple layers. It is also more computationally efficient. Other ReLU generalizations, such as Leaky ReL or Parametric ReLU (PReLU) are also commonly used. However, sigmoid continues to be used as a gating function in recurrent networks to maintain values between 0 and 1, hence controlling what passes through a node (Goodfellow et al. 2016). The hyperbolic tangent (tanh) activation function is also commonly used in recurrent networks, keeping the values that pass through a node between − 1 and 1 (Goodfellow et al. 2016).

#### Learning techniques

**Fig. 5:**
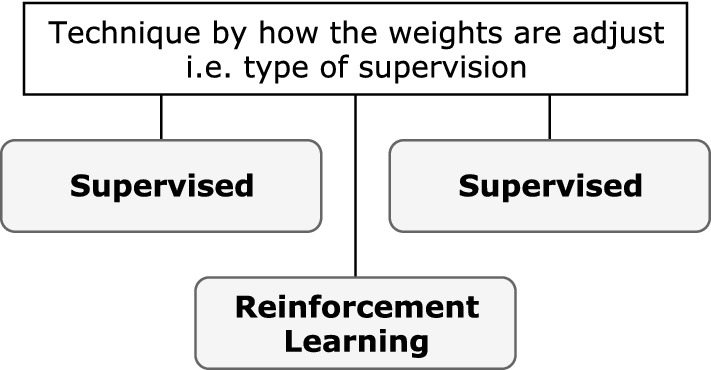
Supervision-based learning technique

**Fig. 6:**
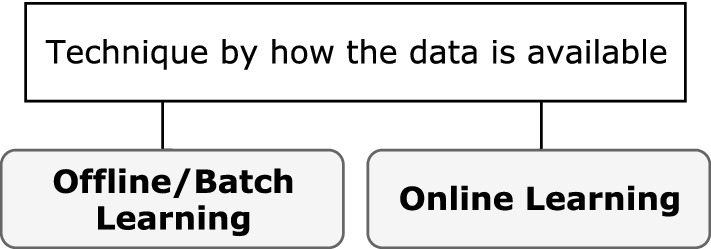
Learning technique based on data availability

In the ANN context, learning refers to the way a network’s parameters adapt according to the input data. Typically, the learning technique is based on how weights are adjusted in the network and how data is made available to the network (Figs. 5, 6).

- Technique based on weight adjustment: The most common learning technique category, this technique is based solely on how weights are adjusted across an iterative process and is dependent on the type of supervision available to the network during the training process. The different types are supervised, unsupervised (or self-organized), and reinforcement learning.
- Technique based on data availability: When categorized according to how data is presented to the network, the learning technique can be considered offline or online. This technique might be chosen because the complete data are not available for training in one batch. This could be because either data are streaming or a concept in the data changes at intervals, requiring the data to be processed in specific time windows. Another reason could be that the data are too large to fit into the memory, demanding processing in multiple smaller batches.

Techniques based on supervision are most common for DL (and indeed DL), with increasing studies adopting batch learning approaches. Nonetheless, the primary architecture of DL networks is not exclusive to one technique category; instead, it is typical to find a mix of both, i.e., offline supervised learning and online reinforcement learning. Unless otherwise specified, it can be assumed that the technique is offline/batch learning. For example, *supervised learning* refers to *offline supervised learning* unless it is specified as online. The key point is that each supervision-based technique can be further categorized according to data availability.

#### Network architecture

**Fig. 7:**
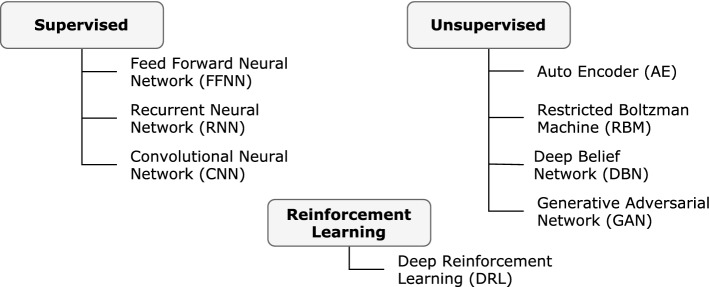
Taxonomy of deep learning architecture used in stock market applications

The architecture of an ANN importantly contributes to the ways that it is organized. Network inputs depend solely on training data, and, for the most part, the output represents a function of the expected output. The layers between the input and output are mostly a design decision that depends largely on the network architecture, which is based on a typical neural network’s system of multiple connections. Numerous ANN architectures exist across various domains, including communication systems and healthcare (Aceto et al. 2019; O’Shea and Hoydis 2017; Xiao 2021), with the stock market applications this survey considers adopting even more derivative architectures with easily identifiable and well-known foundations. Figure 7 presents these architectures and their common categorizations based on how they learn weight parameters). The following section describes their differences.

The learning techniques based on these architectures can be either *discriminative* or *generative*. A discriminative model discriminates between different data classes by learning the boundaries between them or the conditional probability distribution $p(y|x)$; meanwhile, a generative model learns the distribution of individual classes or joint probability distribution $p(x,y)$ (Hinton 2017). Although most traditional ANN architectures are discriminative, *autoencoders* and *BoltzMann machine* are considered generative. In a *Generative Adversarial Network* (Hinton 2017), the two techniques are combined in a novel adversarial manner.

##### Feed-forward neural networks

**Fig. 8:**
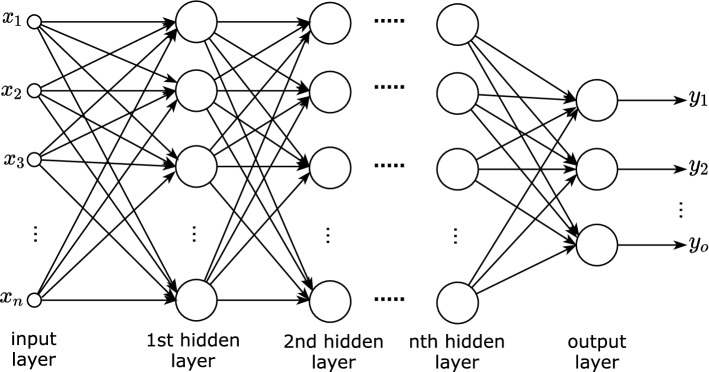
n-layer feed-forward neural network (Castro 2006)

Comprising multiple neurons connected in layers, DL architectures use FFNN widely. Figure 8 presents the architecture of an FFNN. It comprises an *input layer*, representing the input example, one or more *hidden layers*, and an *output layer* (Goodfellow et al. 2016).

Although Goodfellow et al. (2016) suggest that “a single layer is sufficient to represent a function”, hey also recommend deeper layers for better generalization. Ideally, the number of hidden layers should be decided for the specific task via experimentation. The input layer comprises a feature vector representing the input example that is fed to the first hidden layer. The hidden layer(s) and the output layer comprise multiple neurons, each with a vector of weights of the same size as the input, as well as a bias value. Within the layers, each neuron’s output becomes the input for the next layer, until, finally, the output layer uses the final activation to represent the model’s prediction.

Broadly, this process aims to derive a generalization about the weights and biases associated with each neuron in the network, that is, derive generalizable values of ${\mathbf {w}}, b$ to compute $z = \sum \mathbf {wx}+ b$ for each neuron (with input ${\mathbf {x}}$) in the network. Using an iterative training process of forward and backward propagation over multiple examples (training data), each layer’s activations are propagated forward across the network, and the error rate is propagated back to the first hidden layer. Following the learning process, the network (model) can then be used to predict unseen/untested examples.

##### Recurrent neural network

Recurrent Neural Network (RNN) are a special type of neural network that keeps a representation of the previously seen input data. These networks are ideal for processes where the temporal or sequential order of the input example is relevant (Goodfellow et al. 2016).

**Fig. 9:**
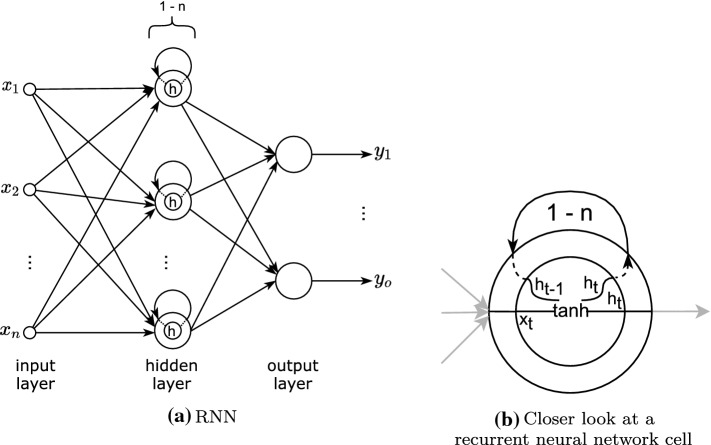
RNN (Goodfellow et al. 2016)

The recurrence is represented as a loop in each neuron, as Fig. 9 shows, allowing one or more passes of the same input, with the network maintaining a state representation of each pass. Following the specified number of passes, the final state is transmitted as output parameters. This means that RNN allow the possibility of inputs and outputs of variable length. That is, given the loop’s flexibility, the architecture can be constructed to be one-to-one, one-to-many, many-to-one, or many-to-many.

**Fig. 10:**
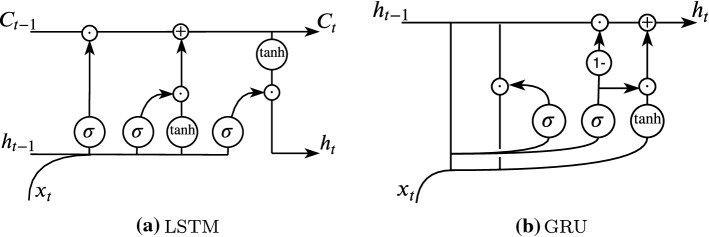
LSTM & GRU (Goodfellow et al. 2016)

However, typical RNN, make it difficult for the hidden state to retain information over a long period. That is, they have a short memory due to the gradient becoming smaller and smaller as it is propagated backward in time steps across the recurring loop, a phenomenon known as *vanishing gradient*. This means that for temporal data, in which the relevant relationship between data points occurs over a lengthy period, a typical RNN model is not ideal. Thus, other versions of RNN have been formulated, with the most frequently used approaches being Long Short-term Memory (LSTM) lstm and Gated Recurrent Unit (GRU) (Goodfellow et al. 2016). The architectures discussed can largely reduce the vanishing gradient effect by maintaining a cell state via additive updates rather than just the RNN hidden state with product updates (Fig. 10).

##### Convolutional neural networks

Another network architecture type that has gained substantial popularity, especially for analyzing digital images, is CNN (Goodfellow et al. 2016). The reason is that CNN can simplify large amounts of pixel density, vastly reducing the number of parameters to work with, making the ANN highly efficient. Unlike more conventional ANN, in which the input is represented as a feature vector, CNN represent the input as a matrix, which they use to generate the first *convolutional layer*.

**Fig. 11:**
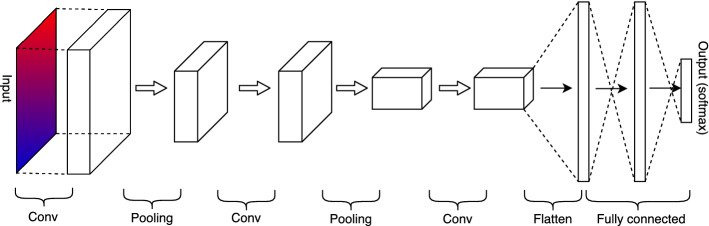
Architecture of a convolutional neural network (Goodfellow et al. 2016)

A typical CNN will contain one or more convolutional layers, each connected to its respective *pooling layer*. Figure 11 provides a simple representation of such a network.

##### Autoencoder

Autoencoders are unsupervised ANN that efficiently encode input data, a process known as *latent representation* or *encoding*. This process involves using input data as a feature vector and attempting to reconstruct the same data using fewer nodes than the input (Goodfellow et al. 2016). As such, autoencoders are frequently used for dimensionality reduction.

**Fig. 12:**
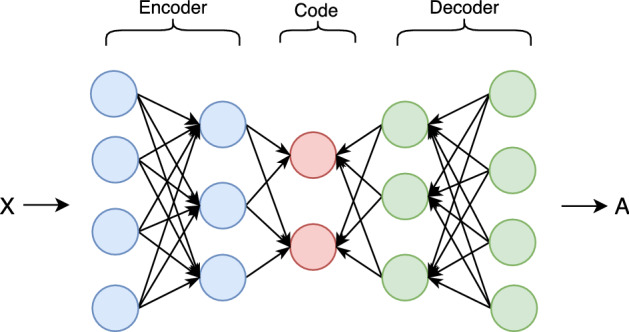
A simple Autoencoder (Goodfellow et al. 2016)

As Fig. 12, shows, an autoencoder’s architecture imposes a bottleneck for encoding the input representation. A decoder layer subsequently reproduces an output to represent the reconstructed input. In so doing, it learns a representation of the input data while ignoring the input noise. The encoder’s representation of the transformed input is referred to as the emphcode, code, and it is the internal or hidden layer of the autoencoder. The decoder subsequently generates the output from the code.

Autoencoders are commonly used in stock market data for their dimension reduction functionality (Chen et al. 2018a; Chong et al. 2017) to avoid dimensionality curse (Soleymani and Paquet 2020). This is an important consideration for stock market data, where there is value in network simplicity without losing important features. In Soleymani and Paquet (2020), a restricted stacked autoencoder network reduces an 11 feature set to a three feature set before it is fed into a CNN architecture in a deep reinforcement learning framework called *DeepBreath*. This enables an efficient approach to a portfolio management problem in a setting that combines offline and online learning. Elsewhere, (Hu et al. 2018a) combines CNN and autoencoder architectures in its *Convoluted Autoencoder (CAE)* to reduce candlestick charts to numerical representations to improve stock similarity.

##### Deep Reinforcement Learning

**Fig. 13:**
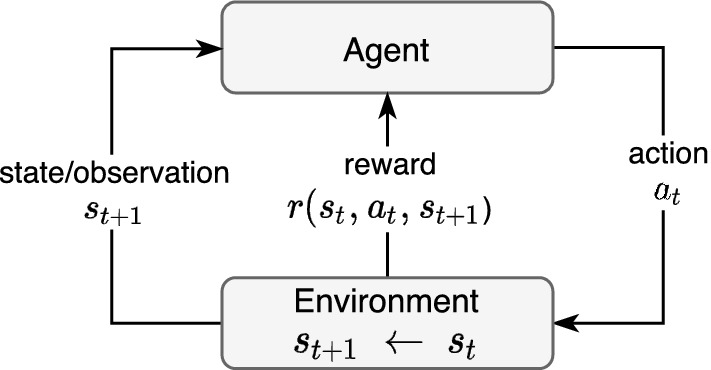
Reinforcement Learning (François-Lavet et al. 2018)

Unlike supervised and unsupervised learning, in which all learning occurs within the training dataset, a *Reinforcement Learning (RL)* problem is formulated as a discrete-time stochastic process. The learning process interacts with the *environment* via an iterative sequence of actions, state transitions, and rewards, in a bid to maximize the cumulative reward (François-Lavet et al. 2018). The future state depends only on the current state and action, meaning it learns using a trial-and-error *reinforcement* process in which an *agent* incrementally obtains experience from its environment, thereby updating its current state (Fig. 13). The action to take (from the action space) by the agent is defined by a *policy*.

It is common to see a RL system formulated as a Markov decision process Markov decision process (MDP) in which the system is fully observable, i.e., the state of the environment is the same as the observation that the agent perceives (François-Lavet et al. 2018). Furthermore, RL can be categorized as *model-based* or *model-free* (Russell and Norvig 2010).

- *Model-based reinforcement learning* The agent retains a transition model of the environment to enable it to select actions that maximize the cumulative utility. The agent learns a *utility function* that is based on the total rewards from a starting state. It can either start with a known model (i.e., chess) or learn by observing the effects of its actions.
- *Model-free reinforcement learning* The agent does not retain a model of the environment, instead focusing on directly learning how to act in different states. This could be via either an *action-utility function* (Q-learning) that learns the utility of taking an action in a given state or a *policy-search* in which a *reflex agent* directly learns to map policy, $\pi (s)$, from different states to corresponding actions.

Deep Reinforcement Learning (DRL) is a deep representation of RL that can be model-based, model-free, or a combination of the two (Ivanov and D’yakonov 2019). The stock market can be considered to feature an DRL characteristic, with past states well-encapsulated in current states and events and the only requirement for future states being the current state. For this reason, DRL is a particularly popular approach for modern quantitative analysis of the stock market. Applications of DRL in these scenarios vary from profitable/value stock selection or portfolio allocation strategy (Wang et al. 2019b; Li et al. 2019) to simulating market trades in a bid to develop optimal liquidation strategy (Bao and Liu 2019).

### Using deep learning in the stock market

In Section 3.1, we considered what DL is and discussed certain specific DL architectures that are commonly used in stock market applications. Although we referred to certain specific uses of these network types that are employed in the stock market, it is important to note that all of the architectures mentioned are also commonly used for other applications. However, some specific considerations must be kept in mind when the stock market is the target. These range from the model’s composition to backtesting and evaluation requirements and criteria. Some of these items do not correspond to a traditional ML toolbox but are crucial to stock market models and cannot be ignored, especially given the monetary risks involved.

This section first discusses the specifics of modeling considerations for stock market applications. It also discusses backtesting as an integral part of the process, and details some backtesting methodology. This is followed by a review of the different evaluation criteria and evaluation types.

#### Modeling considerations

When training an ML model for most applications, we consider how *bias* and *variance* affect the model’s performance, and we focus on establishing the tradeoffs between the two. Bias measures how much average model predictions differ from actual values, and variance measures the model’s generalizability and its sensitivity to changes in the training data. High degrees of bias suggest underfit, and high levels of variance suggest overfit. It is typical to aim to balance bias and variance for an appropriate model fit that can be then applied to any unseen dataset, and most ML applications are tuned and focused accordingly.

However, in financial applications, we must exceed these to avoid some of the following pitfalls, which are specific to financial data.

##### Sampling intervals

Online ML applications typically feature sampling windows in consistent chronological order. While this is practical for most streaming data, it is not suitable for stock market data and can produce substantial irregularities in model performance. As Fig. 2 demonstrates, the volume of trade in the opening and closing period is much higher than the rest of the day for most publicly available time-based market data. This could result from pre-market or after-hours trading and suggests that sampling at a consistent time will inadvertently undersample the market data during high-activity periods and undersample during low-activity periods, especially when modeling for intraday activities.

A possible solution is using data that has been provided in ticks, but these are not always readily available for stock market data without significant fees, potentially hindering academic study. Tick data can also make it possible to generate data in alternative bars, such as tick or volume bars, significantly enhancing the model performance. Notably, (Easley et al. 2012) uses the term *volume clock* to formulate volume bars to align data sampling to volume-wise market activities. This enables high-frequency trading to have an advantage over low-frequency trading.

##### Stationarity

**Fig. 14:**
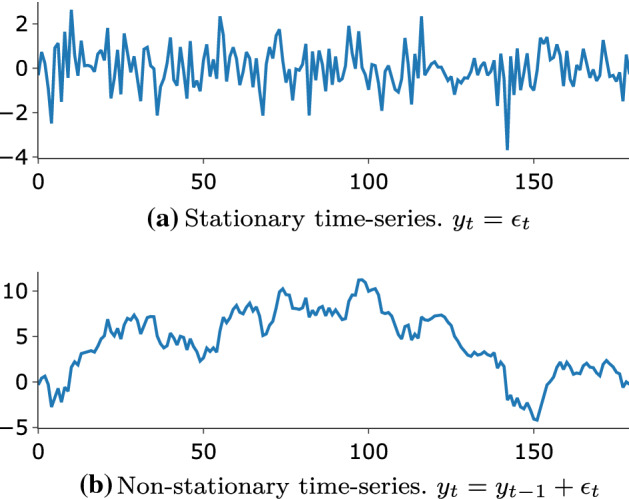
Time-series for the same value of $\epsilon _t \sim {\mathcal {N}}(0,1)$

Time-series data are either stationary or non-stationary. Stationary time-series data preserve the statistical properties of the data (i.e., mean, variance, covariance) over time, making them ideal for forecasting purposes (de Prado 2018). This implies that spikes are consistent in the time series, and the distribution of data across different windows or sets of data within the same series remains the same. However, because stock market data are non-stationary, statistical properties change over time and within the same time series. Also, trends and spikes in non-stationary time series are not consistent. By definition, such data are difficult to model because of their unpredictability. Before any work on such data, it is necessary to render them as stationary time series (Fig. 14).

A common approach to converting non-stationary time series to stationary time series involves differencing. This can involve either computing the difference between conservative observations or, for seasonal time series, the difference between previous observations of the same season. This approach is known as *integral differencing*, with (de Prado 2018) discussing *fraction differencing* as a memory-preserving alternative that produces better results.

##### Backtesting

In ML, it is common to split data into training and testing sets during the modeling process. Given the goal of this exercise is to determine the accuracy or evaluate performance in some other way, it follows that adhering to such a conventional approach is appropriate. However, when modeling for the financial market, performance is measured by the model’s profitability or volatility of the model. According to Arnott et al. (2018), there should be a checklist or *Protocol* that mandates that ML research include the goal of presenting proof of positive outcomes through backtesting.

Opacity and bias in AI systems represent two of the overarching debates in AI ethics (Müller 2020). Although a significant part of the conversation concerns the civil construct, it is clear that the same reasoning applies to other economic and financial AI applications. For example, (Müller 2020) raises concerns about statistical bias and the lack of due process and auditing surrounding using ML for decision-making. This relates to conversations about honesty in backtesting reports and the selection bias that typically affects academic research in the financial domain (Fabozzi and De Prado 2018).

In the context of DL in the stock market, backtesting involves building models that simulate trading strategy using historical data. This serves to consider the model’s performance and, by implication, helps to discard unsuitable models or strategies, preventing selection bias. To properly backtest, we must test on unbiased and sufficiently representative data, preferably across different sample periods or over a sufficiently long period. This positions backtesting among the most essential tools for modeling financial data. However, it also means it is among the least understood in research (de Prado 2018).

When a backtested result is presented as part of a study, it demonstrates the consistency of the approach across various time instances. Recall that *overfitting* in ML describes a model performing well on training data but poorly on test or unseen data, indicating a large gap between the training error and the test error (de Prado 2018). Thus, when backtesting a model on historical data, one should consider the issue of *backtest overfitting*, especially during *walk-forward backtesting* (de Prado 2018).

**Fig. 15:**
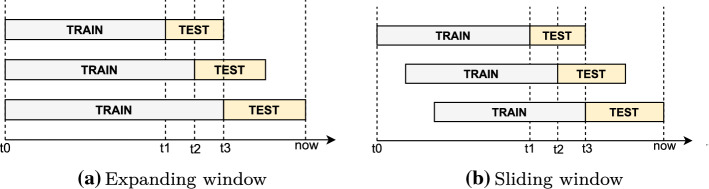
Backtesting strategies

Walk-forward is the more common backtesting approach and refers to simulating trading actions using historical market data—with all of the actions and reactions that might have been part of that—in chronological time. Although this does not guarantee future performance on unseen data/events, it does allow us to evaluate the system according to how it would have performed in the past. Figure 15 shows two common ways of formulating data for backtesting purposes. Formulating the testing process in this manner removes the need for cross-validation because training and testing would have been evaluated across different sets. Notably, traditional K-fold cross-validation is not recommended in time series experiments such as this, especially when the data is not Independent and Identically Distributed (IID) (Bergmeir and Benítez 2012; Zaharia et al. 2010).

Backtesting must be conducted in good faith. For example, given backtest overfitting means that a model is overfitted to specific historical patterns, if favorable results are not observed, researchers might return to the model’s foundations to improve generalizability. That is, researchers are not expected to fine-tune an algorithm in response to specific events that might affect its performance. For example, consider overfitting a model to perform favorably in the context of the 1998 recession, and then consider how such a model might perform in response to the 2020 COVID-19 market crash. By backtesting using various historical data or over a relatively long period, we modify our assumptions to avoid misinterpretations.

##### Assessing feature importance

In discussing backtesting, we have discussed why we shouldn’t selectively “tune” a model to specific historical scenarios to achieve a favorable performance to challenge the usefulness of the knowledge gained from the model’s performance in such experiments. *Feature Importance* becomes relevant here. Feature importance enables the measurement of the contribution of input features to a model’s performance. Given neural networks are typically considered “black-box” algorithms, the movement around explanation AI contributes to the interpretation of the output of the network and understanding of the importance of the constituent features, as observed in the important role of *Feature Importance Ranking* in Samek et al. (2017), Wojtas and Chen (2020). Unlike traditional ML algorithms, this is a difficult feat for ANN models, typically requiring a separate network for the feature ranking.

#### Model evaluation

Machine learning algorithms use evaluation metrics such as accuracy and precision. This is because we are trying to measure the algorithm’s predictive ability. Although the same remains relevant for ML algorithms for financial market purposes, what is ultimately measured is the algorithm’s performance with respect to returns or volatility. The works reviewed include various performance metrics that are commonly used to evaluate an algorithm’s performance in the financial market context.

Recall that in Sect. 3.2.1 emphasized the importance of avoiding overfitting when backtesting. It is crucial to be consistent with backtesting different periods and to be able to demonstrate consistency across different financial evaluations of models and strategies. *Returns* represents the most common financial evaluation metric for obvious reasons. Namely, it measures the profitability of a model or strategy (Kenton 2020). It is commonly measured in terms of rate during a specific window of time, such as day, month, or year. It is also common to see returns annualized over various years, which is known as *Compound Annual Growth Rate (CAGR)*. When evaluating different models across different time windows, higher returns indicate a better model performance.

However, it is also important to consider *Volatility* because returns alone do not relay the full story regarding a model’s performance. Volatility measures the variance or how much the price of an asset can increase or decrease within a given timeframe (Investopedia 2016). Similar to returns, it is common to report on daily, monthly, or yearly volatility. However, contrary to returns, lower volatility indicates a better model performance. The The Volatility Index (VIX), a real-time index from the Chicago Board Options Exchange (CBOE), is commonly used to estimate the volatility of the US financial market at any given point in time (Chow et al. 2021). The VIX measures the US stock market volatility based on its relative strength compared to the S &P 500 index, with measures between 0 and 12 considered low, measures between 13 and 19 considered normal, and measures above 20 considered high.

Building on the information derived from returns and volatility, the *Sharpe ratio* enables investors to identify little-to-no-risk investments by comparing investment returns with risk-free assets such as treasury bonds (Hargrave 2019). It measures average returns after accounting for risk-free assets per volatility unit. The higher the Sharpe ratio, the better the model’s performance. However, the Sharpe ratio features the shortcoming of assuming the data’s normal distribution due to the upward price movement. The*Sortino ratio* can mitigate against this, differing by using only the standard deviation of the downward price movement rather than the full swing that the Sharpe ratio employs.

Other commonly used financial metrics are *MDD* and the *Calmar ratio*, both of which are used to assess the risk involved in an investment strategy. Maximum drawdown describes the difference between the highest and lowest values between the start of a decline in peak value to the achievement of a new peak value, which indicates losses from past investments (Hayes 2020). The lower the MDD, the better the strategy, with zero value suggesting zero loss in investment capital. The Calmar ratio measures the MDD adjusted returns on capital to gauge the performance of an investment strategy. The higher the Calmar ratio, the better the strategy.

Another metric considered important by the works reviewed was VaR, which measures risk exposure by estimating the maximum loss of an investment over time using historical performance (Harper 2016).

Meanwhile, other well-known non-financial ML metrics commonly used are based on the accuracy of a model’s prediction. These metrics are calculated in terms of either the following *confusion matrix* or in terms of the difference between the derived and observed target values.

**Table:** 

		Predicted		
		Positive	Negative	Total
Actual	Positive	*TP*	*FP*	*P*
	Negative	*FN*	*TN*	*N*
	Total	$P'$	$N'$	$P+N$

True Positive (TP) and True Negative (TN) are the correctly predicted positive and negative classes respectively. Subsequently, False Positive (FP) and False Negative (FN) are the incorrectly predicted positive and negative classes (Han et al. 2012).

The evaluation metrics in Table 7 are expected to be used as complementary metrics to the primary and more specific financial metrics in Table 6. This is because the financial metrics can evaluate various investment strategies in the context of backtested data, which the ML metrics are not designed for. Section 4 demonstrates how these different evaluation metrics are combined across the works of literature that we reviewed (Table 7).

**Table 6:** Financial evaluation metrics

Evaluation	Description	Formula
Returns	Total amount gained or lost within a specific investment period, typically measured as a percentage of the original investment known as Rate of Returns (RoR) (Kenton 2020). This could also be the absolute total profit or loss for the investment period	$\displaystyle \frac{V_f - V_i}{V_i} * 100$.
Compound annual growth rate (CAGR)	The ROR for investment over a number of years, with returns re-invested yearly (Murphy 2019). *n*: Number of years	$\displaystyle \left( \frac{V_f}{V_i}\right) ^\frac{1}{n} - 1$
Volatility	Degree of variation in asset or total portfolio value (Investopedia 2016). $\sigma$: Standard deviation of returns; *T*: *Time Horizon* or number of holding period	$\displaystyle \sigma \sqrt{T}$
Sharpe ratio	Measures performance in comparison with a risk-free asset, with adjustments for volatility or total risk (Hargrave 2019). $R_p$: Average portfolio returns; $r_f$: Risk-free (i.e., treasury bonds) returns; $\sigma _p$: standard deviation of a portfolio’s excess returns	$\displaystyle \frac{R_p - r_f}{\sigma _p}$
Sortino ratio	A modification of the Sharpe Ratio that differentiates harmful volatility from overall volatility (Kenton 2019). $\sigma _d$: standard deviation of portfolio’s negative returns, i.e., returns that fall below a user-defined threshold	$\displaystyle \frac{R_p - r_f}{\sigma _d}$
Maximum drawdown (MDD)	Measures the decline of a return from a peak before a new peak that is at least equal to the old peak is achieved (Hayes 2020). This is used to compare the riskiness of different models or strategies. $V_t$: Trough value; $V_p$: Peak value	$\displaystyle \frac{V_t - V_p}{V_p}$
Calmar ratio	Risk-adjusted returns (Will Kenton 2020).	$\displaystyle \frac{V_f - V_i}{MDD}$
Value-at-risk (VaR) threshold	Estimate (as threshold) of maximum loss for an investment over time (Harper 2016). $E_r$: Expected returns; $z_i$: *z*-score of confidence interval; $\sigma _p$: Standard deviation of portfolio; $V_p$: Value of portfolio	$\displaystyle \left[ E_r - \left( z_i * \sigma \right) \right] * V_p$

**Table 7:** Machine learning evaluation metrics

Evaluation	Description	Formula
Accuracy	The percentage of the correctly predicted classes.	$\displaystyle \frac{TP + TN}{P + N}$
Error rate	The percentage of incorrectly predicted classes. Also computed as $1 - accuracy$.	$\displaystyle \frac{FP + FN}{P + N}$
Recall	Ratio of true positive classes; also known as measure of exactness or sensitivity.	$\displaystyle \frac{TP}{P}$
Precision	Ratio of positive predictions; also known as measure of completeness.	$\displaystyle \frac{TP}{TP + FP}$
F-score	Harmonic mean of recall and precision.	$\displaystyle \frac{2 * precision * recall}{precision + recall}$
Weighted F-score	Weighted measure of recall and precision. $\beta < 1$ assigns more weight to precision, while $\beta > 1$ assigns more wait to recall.	$\displaystyle \frac{(1+\beta ^2) * precision * recall}{\beta ^2 * precision + recall}; \beta > 0$
Mean absolute error (MAE)	Average of the absolute difference between the predicted values and the actual values.	$\displaystyle \frac{1}{n}\sum ^n_{i=1}|y_i - {\hat{y}}_i|$
Mean absolute percentage error (MAPE)	Average of the percentage errors.	$\displaystyle \frac{100}{n}\sum ^n_{i=1} \frac{y_i - {\hat{y}}_i}{y_i}$
Mean square error (MSE)	Average of the squared difference between the predicted values and the actual values.	$\displaystyle \frac{1}{n}\sum ^n_{i=1}\left( y_i - {\hat{y}}_i \right) ^2$

#### Lessons learned

This section has reviewed different types of deep ANN architectures that are commonly used in the stock market literature considering DL. The ANN landscape in this context is vast and evolving. We have focused on summarizing these architectures on the basis of their recurrence across different areas of specialization within the stock market. Explicitly recalling the architectures used should assist explanations of their usage as we proceed to our findings in Sect. 4.

We have similarly detailed the expectations of modeling for the financial market and how these differ from the traditional ML approach, an important consideration for the rest of the survey. That is, although it is worthwhile applying methodologies and strategies across different areas of a discipline to advance scientific practice, we should endeavor to also attend to established practice and the reasoning behind that practice. This includes also understanding the kinds of metrics that should be used. In conducting this survey, we identified several works that used only ML metrics, such as accuracy and F-score, as evaluation metrics (Ntakaris et al. 2019; Lee and Yoo 2019; Kim and Kang 2019; Passalis et al. 2019; Ganesh and Rakheja 2018). Although this might be ideal for complementary metrics, the performance of an algorithm or algorithmic strategy must ultimately be relevant to the study domain. By more deeply exploring intra-disciplinary research in the computer science field, we begin to understand the space we open up and the value we confer in the context of established processes.

By highlighting various considerations and relevant metrics, we trust that we have facilitated computer science research’s exploration of ideas using stock market data and indeed contributed to the research in the broader econometric space. The next section presents this survey’s culmination, discussing how the findings relate to the previously discussed background and attempting to answer the study’s research questions and demonstrating the criteria employed to shortlist the literature reviewed.

## Survey findings

### Research methodology

This research work set out to investigate applications of DL in the stock market context by answering three overarching research questions:

#### Question 1

What current research methods based on deep learning are used in the stock market context?

#### Question 2

Are the research methods consistent with real-world applications, i.e., have they been backtested?

#### Question 3

Is this research easily reproducible?

Although many research works have used stock market data with DL in some form, we quickly discovered that many are not easily applicable in practice due to how the research has been conducted. Although we retrieved over 10,000 works^1^, by not being directly applicable, most of the experiments are not formulated to provide insight for financial purposes, with the most common formulation being as a traditional ML problem that assumes that it is sufficient to break the data into training and test sets.

Recall that we categorized learning techniques by data availability in Sect. 3.1.2. When the complete data are available to train the algorithm, it is defined as *offline* or *batch* learning. When that is not the case, and it is necessary to process the data in smaller, sequential phases, as in streaming scenarios or due to changes in data characteristics, we categorize the learning technique as *online*. Although ML applications in the stock market context are better classified as online learning problems, surprisingly, very few research papers approach the problem accordingly, instead mostly approaching it as an offline learning problem, a flawed approach (de Prado 2018).

To apply this approach to financial ML research for the benefit of market practitioners, the provided insight must be consistent with established domain norms. One generally accepted approach to achieving this is backtesting the algorithm or strategy using historical data, preferably across different periods (Bergmeir and Benítez 2012; Institute 2020). Although Sect. 3.2.1 discussed backtesting, we should re-iterate that backtesting does not constitute a “silver bullet” or a method of evaluating results. However, it does assist evaluation of the performance of an algorithm across different periods. Financial time-series data are not IID, meaning the data distribution differs across different independent sets. This also means that there is no expectation that results across a particular period will produce similar performances in different periods, no matter the quality of the presented result. Meanwhile, the relevant performance evaluation criteria are those that are financially specific, as discussed in Sect. 3.2.2. To this end, we ensured that the papers reviewed provide some indication of consideration of backtesting. An ordinary reference sufficed, even if the backtested results are not presented.

We used Google Scholar (Google 2020) as the search engine to find papers matching our research criteria. The ability to search across different publications and the sophistication of the query syntax (Ahrefs 2020) was invaluable to this process. While we also conducted spot searches of different publications and websites to validate that nothing was missed by our chosen approach, the query results from Google Scholar proved sufficient, notably even identifying articles that were missing from the results of direct searches on publication websites. We used the following query to conduct our searches:

“deep learning” AND “stock market” AND (“backtest” OR “back test” OR “back-test”)

This query searches for publications including the phrases “deep learning”, “stock market”, and any one of “backtest”, “back test” or “back-test”. We observed these three different spellings of “backtest” in different publications, suggesting the importance of catching all of these alternatives. This produced 185 results^2^, which include several irrelevant papers. For validation, we searched using Semantic Scholar (Scholar 2020), obtaining approximately the same number of journal and conference publications. We chose to proceed with Google Scholar because Semantic Scholar does not feature such algebraic query syntax, requiring that we search for the different combinations of “backtest” individually with the rest of the search query.

**Table 8:** Quantifying papers by publication and year of publication

Publisher	Count	Year	Count
IEEE	9	2018	6
arXiv	8	2019	10
SSRN	5	2020	19
Elsevier	3		
ACM	2		
MDPI	2		
Springer	2		
IOP Publishing	1		
Wiley	1		
IJCAI	1		
Institutional Investor Journals	1		

The search query construct provided us with the starting point for answering research questions (1) and (2). Then, we evaluated the relevance to the research objective of the 185 publications and considered how each study answered question (3). We objectively reviewed all query responses without forming an opinion on the rest of their experimental procedure with the rationale that addressing the basic concerns of a typical financial analyst represents a good starting point. Consequently, we identified only 35 papers as relevant to the research objective. Table 8 quantifies the papers reviewed by publication and year of publication. It is interesting to observe the non-linear change in the number of publications over the last 3 years as researchers have become more conscious of some of these considerations

**Table 9:**
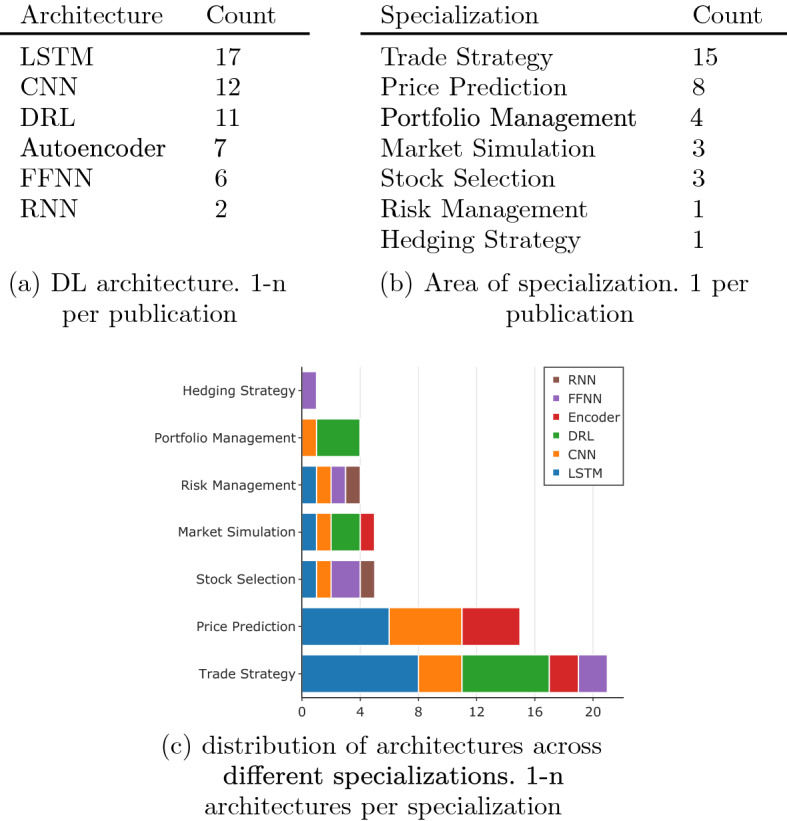
Quantifying the architectures and _elds considered by publications surveyed

### Summary of findings

Section 3.1.3 explained the different architectures of the deep ANN that are commonly used in stock market experiments. Based on the works reviewed, we can categorize the algorithms into the following specializations:

- Trade Strategy: Algorithmically generated methods or procedures for making buying and selling decisions in the stock market.
- Price Prediction: Forecasting the future value of a stock or financial asset in the stock market. It is commonly used as a trading strategy.
- Portfolio Management: Selecting and managing a group of financial assets for long term profit.
- Market Simulation: Generating market data under various simulation *what-if* market scenarios.
- Stock Selection: Selecting stocks in the stock market as part of a portfolio based on perceived or analyzed future returns. It is commonly used as a trading or portfolio management strategy.
- Risk Management: Evaluating the risks involved in trading, to maximize returns.
- Hedging Strategy: Mitigating the risk of investing in an asset by taking an opposite investment position in another asset.

Although a single specialization is usually the primary area of focus for a given paper, it is common to see at least one other specialization in some form. An example is testing a minor trade strategy in price prediction work or simulating market data for risk management. Table 9 illustrates the distribution of the different DL architectures across different areas of specialization for the studies reviewed by this survey. Architectures such as LSTM and DRL are more commonly used because of their inherent temporal and state awareness. In particular, lstm is favorable due to its relevant characteristic of remembering states over a relatively long period, which price prediction and trade strategy applications, in particular, require. Novel use cases (e.g., (Wang et al. 2019b) combine LSTM and RL to perform remarkably well in terms of annualized returns. There are many such combinations in trade strategy and portfolio management, where state observability is of utmost importance.

Although FFNN is seldom used by itself, there are multiple instances of it being used alongside other approaches, such as CNN and RNN. Speaking of CNN, it is surprising how popular it is, considering it is more commonly used for image data. True to its nature, attempts have been made to train models using stock market chart images (Kusuma et al. 2019; Hu et al. 2018a). Given its ability to localize features, CNN is also used with high-frequency market data to identify local time series patterns and extract useful features (Chong et al. 2017). Autoencoders and Restricted Boltzmann machine (RBM) are also used for feature extraction, with the output fed into another kind of deep neural network architecture (Table 10).

**Table 10:** Summary of publications

	A: architecture, B: market(s), C: dataset source, D: reproducibility				
	References	A	B	C	D
Trade strategy	Wang et al. (2019c)	DRL, LSTM	China, US	wind, wrds	No
	Li et al. (2020)	DRL	US	kaggle	No
	Théate and Ernst (2020)	DRL, FFNN	Asia, US, Europe	unspecified	Yes
	Zhang et al. (2020d)	DRL	US	pinnacle	No
	Chakole and Kurhekar (2020)	DRL, FFNN	US, India	yahoo	No
	Wu et al. (2019)	DRL, LSTM	China	tushare	No
	Hu et al. (2018b)	Autoencoder, CNN	UK	unspecified	No
	Lei et al. (2020)	CNN	China	tushare	No
	Chen et al. (2018b)	CNN	Taiwan	apex	No
	Wu et al. (2020)	LSTM	Taiwan	tfe	No
	Koshiyama et al. (2020)	Autoencoder, LSTM	Global	bloomberg	Yes
	Sun et al. (2019)	LSTM	US	ibkr	No
	Silva et al. (2020)	LSTM	Unspecified	unspecified	No
	Wang et al. (2020)	LSTM	China	joinquant	No
	Chalvatzis and Hristu-Varsakelis (2020)	LSTM	US	unspecified	No
Price prediction	Wang et al. (2019a)	Conv-LSTM, RNN	China, US	ibkr	No
	Zhang et al. (2019)	CNN, LSTM	UK, Nordic	lse, etsin	No
	Zhao et al. (2018)	Autoencoder, CNN, LSTM	US	unspecified	No
	Zhang et al. (2020b)	Autoencoder, CNN, LSTM	China	unspecified	No
	Fang et al. (2019)	LSTM	China	private	No
	Baek and Kim (2018)	LSTM	US	yahoo	Yes
	Wang et al. (2018)	CNN	US	unspecified	No
	Zhang et al. (2020c)	Autoencoder	China	unspecified	No
Portfolio management	Liang et al. (2018)	DRL	China	investing, wind	Yes
	Park et al. (2020)	DRL	Korea, US	investing, yahoo	No
	Guo et al. (2018)	DRL, CNN	China	unspecified	Yes
	Wang and Wang (2019)	FFNN	US	bloomberg	No
Market simulation	Maeda et al. (2020)	DRL, LSTM, CNN	Simulated	none	No
	Buehler et al. (2020)	Autoencoder	US	unspecified	Yes
	Raman and Leidner (2019)	DRL	US	trkd	No
Stock selection	Zhang et al. (2020a)	FFNN	China	unspecified	No
	Amel-Zadeh et al. (2020)	RNN, FFNN	US	wrds	No
	Yang et al. (2019)	CNN, LSTM	China	unspecified	No
Risk management	Arimond et al. (2020)	CNN, FFNN, LSTM, RNN	EU, UK, US	refinitive	No
Hedging strategy	Ruf and Wang (2020)	FFNN	EU, US	optionm, datashop	Yes

*investing* investing.com, *wrds* wrds-www.wharton.upenn.edu, *bloomberg* bloomberg.com, *yahoo* finance.yahoo.com, *kaggle* kaggle.com, *ibkr* interactivebrokers.com, *tfe* taifex.com.tw, *tushare* pypi.org/project/tushare, *optionm* optionmetrics.com, *refinitive* refinitiv.com, *datashop* datashop.deutsche-boerse.com, *trkd* trkd.thomsonreuters.com, *wind* wind.com.cn, *etsin* etsin.fairdata.fi, *lse* londonstockexchange.com, *pinnacle* pinnacledata2.com, *apex* apex.com.tw, *joinquant* joinquant.com

We further examined the evaluation metrics used by the reviewed works. Recall that Sect. 3.2.2 presented the different financial and ML evaluation metrics observed by our review. As Table 11 shows, returns constitute the most commonly used comparison measure for obvious reasons, especially for trade strategy and price prediction; the most common objective is profit maximization. It is also common to see different derivations of returns across different time horizons, including daily, weekly, and annual returns (Wang et al. 2019c; Théate and Ernst 2020; Zhang et al. 2020a).

Although ML metrics such as accuracy and MSE are typically combined with financial metrics, it is expected that the primary focus remains financial metrics; hence, these are the most commonly observed.

**Table 11:** Quantifying evaluation measures used in different specializations

	TS	PP	MS	SS	PM	RM	HS
Returns	13	8	2	2	4	1	0
MDD	8	2	1	2	0	0	0
Sharpe ratio	7	3	1	1	3	0	0
Sortino ratio	3	0	0	0	0	0	0
Calmar ratio	3	0	0	0	0	0	0
Accuracy	3	1	0	2	0	0	0
Volatility	3	0	0	0	0	0	0
Recall	2	1	1	1	0	0	0
Precision	2	2	1	1	0	0	0
F-score	2	1	1	1	0	0	0
VaR threshold	0	0	0	0	0	1	0
MAE	1	1	0	0	0	0	0
MAPE	1	1	0	0	0	0	0
MSE	1	3	0	0	0	0	1

*TS* trade strategy, *PP* price prediction, *MS* market simulation, *SS* stock selection, *PM* portfolio management, *RM* risk management, *HS* hedging strategy

The following observations can be made based on the quantified evaluation metrics presented in Table 11:

- Returns is the most common financial evaluation metric because it can more intuitively evaluate profitability.
- Maximum drawdown and Sharpe ratio are also common, especially for trade strategy and price prediction specialization.
- The Sortino and Calmar ratios are not as common, but they are useful, especially given the Sortino ratio improves upon the Sharp ratio, and the Calmar ratio adds metrics related to risk assessment. Furthermore, neither is computationally expensive.
- For completeness, some studies include ML evaluation metrics such as accuracy and precision; however, financial evaluation metrics remains the focus when backtesting.
- Mean square error is the more common error type used (i.e., more common than MAE or MAPE).

#### Findings: trade strategy

A good understanding of the current and historical market state is expected before making buying and selling decisions. Therefore, it is understandable that DRL is particularly popular for trade strategy, especially in combination with LSTM. The feasibility of using DRL for stock market applications is addressed in Li et al. (2020), which also articulates the credibility of using it for strategic decision-making. That paper compares implementations of three different DRL algorithms with the Adaboost ensemble-based algorithm, suggesting that better performance is achieved by using Adaboost in a hybrid approach with DRL.

The authors of Wang et al. (2019c) address challenges in quantitative financing related to balancing risks, the interrelationship between assets, and the interpretability of strategies. They propose a DRL model called *AlphaStock* that uses LSTM for state management to address the issue. For the interrelationship amongst assets, (Vaswani et al. 2017) proposes a Cross-Asset Attention Network (caan) using an Attention Network. This research uses the buy-winners-and-sell-losers (BWSL) trading strategy and is optimized on the Sharpe ratio, evaluating performance according to profit and risk. The approach demonstrates good performance for commutative wealth, performing over three times better than the market. Although there could be some questions regarding the way the training and test sets were divided, especially given cross-validation was not used, this work demonstrates an excellent implementation of a DL architecture using stock market data.

Elsewhere, (Théate and Ernst 2020) maximizes the Sharpe ratio using a state-of-the-art DRL architecture called the Trading Deep Q-Network (TQDN) and also proposes a performance assessment methodology. To differentiate from the Deep Q-Network (DQN), which uses a CNN algorithm as the base, the TQDN uses an FFNN along with certain hyperparameter changes. This is compared with common baseline strategies, such as buy-and-hold, sell-and-hold, trend with moving average, and reversion with moving average, producing the conclusion that there is some room for performance improvements. Meanwhile, (Zhang et al. 2020d) uses DRL as a trading strategy for futures contracts from the Continuously Linked Commodities (CLC) database for 2019. Fifty futures are investigated to understand how performance varies across different classes of commodities and equities. The model is trained specifically for the output trading position, with the objective function of maximizing wealth. While the literature also includes forex and other kinds of assets, we focused on stock/equities. Other DRL applications include (Chakole and Kurhekar 2020), which combines DRL with FFNN, and (Wu et al. 2019), which combines DRL with LSTM.

Among non-DRL architectures, the most common we observed were CNN and LSTM. In Hu et al. (2018b), Candlestick charts are used as input for a CAE, primarily to capture non-linear stock dynamics, and long periods of historical data are represented as charts. The algorithm starts by clustering stocks by sector and selects top stocks based on returns within each cluster. This procedure outperforms the FTSE 100 index over 2,000 backtested trading days. It would be interesting to observe how this compares to using the numbers directly instead of using the chart representation.

Given Moving Average Convergence/Divergence (MACD) is known to perform worse than expected in a stable market (Lei et al. 2020), uses *uses Residual Network (ResNet)*, a layer-skipping mechanism, to improve its effectiveness. The authors propose a strategy called MACD-KURT, which is based on *ResNet* as an algorithm and Kurtosis as a prediction target. Meanwhile, (Chen et al. 2018b) uses a filterbank to generate 2D visualizations using historical time series data. Fed into CNN for pair trading strategy, this helps to improve accuracy and profitability. It is also common to observe LSTM-based strategies, either for converting futures into options (Wu et al. 2020), in combination with Autoencoders for training market data (Koshiyama et al. 2020), or in more general trade strategy applications (Sun et al. 2019; Silva et al. 2020; Wang et al. 2020; Chalvatzis and Hristu-Varsakelis 2020).

#### Findings: price prediction

The *Random Walk Hypothesis*, popularized by Malkiel (1973), suggests that stock price changes in random ways, similar to a coin toss, precluding prediction. However, because price changes are influenced by factors other than historical price, numerous papers and practical applications combine all of these to attempt to obtain some insight into price movement. Given the temporal nature of buying and selling, the price prediction specialization also requires some degree of historical context. For this reason, RNN and LSTM are, unsurprisingly, often relied on. However, what is surprising is the novel use of CNN for this purpose, either as an independent algorithm or in combination with RNN algorithms.

For example, (Wang et al. 2019a) takes inspiration from RNN applications involving observing repeating patterns in speech and video, proposing Convolutional LSTM-based Variational Sequence-to-Sequence model with Attention (CLVSA) as a hybrid comprising RNN and convoluted RNN. The paper also introduces Kullback-Leibler divergence (KLD) to address overfitting in financial data. This work follows an optimal backtesting method involving training and testing in a sliding windows approach for 8 years. Specifically, from the start of the period, the model is trained on 3 years of data, evaluated on 1 week of data, and tested the following week. Then, the training regimen shifts forward by a week before being repeated until the end of the period. However, there is no indication of whether the model is updated (i.e., online learning) or a net-new model is introduced for each sliding window. The latter is suspected. Nonetheless, the experiment shows that the algorithm produces very high returns. Elsewhere, (Baek and Kim 2018) proposes an LSTM architecture called *ModAugNet* as a data augmentation approach designed to prevent overfitting of stock market data.

Although most algorithms use data from market trades, DeepLOB (Zhang et al. 2019) uses Limit Order Book (LOB) data with Google’s Inception Module CNN to infer local interaction and feed the output to an LSTM model. It uses a CNN filter to capture spatial structure in LOB and LSTM to capture time dependencies, achieving Accuracy/Precision/Recall/F1 in the 60–70% range. The study also performs a minor simulation to test a mock trade strategy using the model’s prediction. It would be interesting to see results on returns based on a full trade strategy or portfolio management.

In another use of LSTM with other architectures (Zhao et al. 2018), incorporates fundamental and technical indicators to create a market attention model featuring a temporal component to learn a representation of the stock market. They propose *MarketSegNet*, a convolutional Autoencoder architecture that uses an image of numerical daily market activities to generate a generic market feature representation. The generated features are subsequently fed into an LSTM architecture to generate the prediction model. The results of such an approach compared with a model using actual numbers would be interesting to consider. Elsewhere, (Zhang et al. 2020b) compares LSTM with two different LSTM hybrid architectures, one using Autoencoder and one using CNN. Although the hybrid versions demonstrate better performance on accuracy tests, one hybrid’s performance is only slightly better than non-hybridized LSTM in terms of Returns/Sharpe Ratio. Meanwhile, (Fang et al. 2019), combines a non-NN Regression model with LSTM, concluding that the hybrid is better than the plain LSTM in terms of accuracy but less stable when backtested.

In terms of non-LSTM architectures (Wang et al. 2018), uses a one-dimensional CNN for price prediction, with the results suggesting that the model can extract more generalized feature information than traditional algorithms. This claims to be the first application of CNN on financial data, with the authors suggesting that their method achieves a significantly higher Sharpe ratio than Support Vector Machines (Support Vector Machines (SVM)), FFNN, and simple buy-and-hold. Furthermore, the work proposes a weighted F-score that assigns priority to the different errors based on how critical they are. It is suggested that the weighted F-score works better than the traditional F-score for financial data. Finally, (Zhang et al. 2020c) achieves a promising performance with a much simpler approach, using an Autoencoder algorithm for feature extraction alone.

#### Findings: portfolio management

Portfolio management represents another specialization area that relies heavily on DRL. In (Liang et al. 2018), three state-of-the-art gameplay and robotics DRL algorithms, namely, Deep Deterministic Policy Gradient (DDPG), Proximal Policy Optimization (PPO), and Policy Gradient (PG), are implemented for portfolio management. The paper also proposes a new training method that improves efficiency and returns in the Chinese stock market. This approach does not produce favorable results, with the authors discovering that their model needs more data to work sufficiently in a bull market. Adjusting the objective function does not help to alleviate the risk, which is deemed too complex. However, it represents one of the earliest works to attempt to properly tackle the problem of conducting DL research using stock market data.

The authors of Park et al. (2020) also use DRL—specifically, Q-Learning—for optimal portfolio management across multiple assets. Departing from a formulated trading process, they use an MDP in which the action space, with respect to size, is the trading direction. They also use a mapping function to derive a reasonable trading strategy in the action space and simulate actions in the space, enabling them to obtain experience beyond the available data. For a baseline comparison, the authors use known strategies, such as buy-and-hold, random selection, momentum (buy improvement in previous or sales decrease in previous, based on priority), and reversion (opposite of momentum). Their experimentation outperforms baseline comparisons in terms of overall returns.

The authors of Guo et al. (2018) propose the Robust Log-Optimal Strategy (RLOS) as part of an ensemble of pattern matching strategies comprising RLOS and DRL (i.e., RLOSRL) for portfolio management. This approach, based on the log optimal (logarithmically optimal rate of returns), approximates log function using Taylor expansion. It was compared with the naïve average and follows the winning strategies as a baseline. Both RLOS and RLOSRL perform better than all other approaches across multiple backtests with consistently impressive returns. Notably, the RLOSRL demonstrates superior performance, potentially significantly due to the state-aware DRL architecture. To help understand the environmental state, (Wang and Wang 2019) uses FFNN with *ResNet* to address overfitting problems associated with noisy financial data, applying the strategy to regime-switching (statistical change in the data series) and concluding that *ResNet* performs better than a regular FFNN.

#### Findings: market simulation

Historical data are very useful and commonly used to evaluate performance over different known states. However, this features the problem that it relies entirely upon history, and the state is fully known and encapsulated into past market and economic events, introducing complications when unknown states or future what-if scenarios must be tested to ensure a robust model performance in such circumstances. Consider, for example, that a SARS-like global pandemic had been predicted for several years before the COVID-19 outbreak of 2020. It would have been useful to know how the market might react before the pandemic. In this context, market data simulation is invaluable.

The authors in Maeda et al. (2020) propose a market DRL framework to help improve the performance of DL algorithms using a combination of DRL and LSTM with simulated market data. By simulating the order books for limit, market, and cancel orders, they are able to maximize returns. This draws upon the premise that because past market actions might not represent a good indicator for the future, it is better to use simulated data for backtesting purposes. Also, specific scenarios can be created using simulated data that does not correspond to past situations, enabling the generation of data for the forecasted circumstance. This combines market simulation with trade strategy specialization. For a baseline, it compares random market actions using the same simulated data, achieving consistently impressive results.

A different approach is taken in Raman and Leidner (2019), which uses 6 weeks of real market data to generate simulated data. The authors use a DRL model to decide on a trading decision (sell, hold, or buy) for the simulated conditions, comparing the algorithm with other baseline strategies and comparing the simulated data with Monte Carlo simulations. It would be interesting to see comparisons with substantially longer time frames. Elsewhere, (Buehler et al. 2020) introduces a financial time series market simulation that relies on a very small amount of training data, using the signature of historical path segments known as “rough paths” (Vaswani et al. 2017; Boedihardjo et al. 2016) in combination with an Autoencoder. Interestingly, the authors conclude that the data generated are not significantly better than the market data and are useful for test purposes but not for real applications.

#### Findings: stock selection

The stock selection problem is at the core of most, if not all, stock market specializations. This represents a hard problem that some deem impossible to solve. According to Malkiel (1973), a group of monkeys throwing darts at a financial page will perform equally as well as experts in the task of stock selection. Nonetheless, this has not stopped researchers exploring the problem. Although the research focus usually exceeds the singular action of selecting the stock, few studies really emphasize either this or the reasoning behind it.

The authors of Zhang et al. (2020a) use a feature selection technique called *DoubleEnsemble* to identify key features from stock market data. This involves training sub-models [FFNN or gradient boosting ensembles (Zhang et al. 2020a)] with weighted features to alleviate overfitting problems and stabilize them to learn with noisy financial data. To prevent stability issues and incurring huge costs by retraining models after feature removal, as traditional approaches do, a shuffling-based feature selection method has been proposed. This means that different feature sets are trained across different sample sets, and loss is measured as indicated by the missing feature. The authors backtest by hedging on a position based on model prediction, with the results showing significantly improved returns and Sharpe ratio in the context of China’s A-share market. It would be interesting to see how this compares to traditional feature reduction methods, such as Principal Component Analysis [(Principal Component Analysis (PCA)], in terms of performance, compute cost, and returns.

More sophisticated architectures have also been used. For instance, (Yang et al. 2019) uses CNN and LSTMfor a stock trading strategy based on stock selection. Their proposal builds features directly from the Chinese market, and a purchase is made from the model’s prediction based on a projected profit of ≥ 0.14%. The models perform significantly better than the baseline of CSI300 in the Chinese market, which is impressive considering transaction fees are included. Interestingly, a CNN-based architecture outperforms an LSTM-based architecture. This study features the drawback of not providing a comparison with a simple, baseline strategy, such as buy and hold.

Rather than constructing features using market data alone (Amel-Zadeh et al. 2020), bases its predictions entirely on existing financial statement data (from Compustat), comparing RNN and LSTM with non-DL algorithms, such as random forest and regression. These experiments achieve a mild, slightly-better-than-chance prediction rate of 53–59%, with the random forest model outperforming the DL algorithms in terms of returns. There is no evidence that lagged-time fundamentals are included as a factor in the feature engineering procedure.

#### Findings: risk management

Aiming to minimize risk to maximize returns, risk management represents an important specialization that must be incorporated into other strategies. However, our findings reveal that limited attention is focused on this specialization. Nonetheless, the recent market crash of 2020, caused primarily by the COVID-19 pandemic (Wikipedia 2020a), is likely to renew interest in this line of research, with at least one study already motivated by these events.

That study, (Arimond et al. 2020), compares FFNN, temporal CNN, and LSTM algorithms with the Hidden Markov Model (Hidden Markov Model (HMM)) to estimate the VaR threshold. A VaR breach is reached when portfolio returns fall below the threshold. The model is trained to estimate the probability of regime change, referred to as regime-switching. This is commonly modeled as the change in market condition from a bull market (trending up) to a bear market (trending down). By estimating the moment of the VaR breach, it is possible to mitigate the risk to the portfolio.

#### Findings: hedging strategy

Similar to risk management, the hedging strategy specialization does not feature an extensive work of literature that fits our survey of backtested DL research in the stock market context. The authors of Ruf and Wang (2020) propose HedgeNet for generating a hedging strategy using FFNN over one period. Rather than predicting an estimate for option price and using that as the hedging strategy, a hedging ratio is predicted directly from the FFNN, the main metric of interest. This aligns with a recommendation from Bengio (1997).

Considering hedging strategies rely on training pairs of an asset at opposite positions, it would be interesting to see applications of state-conscious algorithms, such as DRL or LSTM, applied in this context.

**Table 12:** Highlights of and problems with publications reviewed

Ref.	Highlights/pros	Problems/cons
*Trade strategy*		
Wang et al. (2019c)	Clear implementation of DRL and LSTM with adequate historical data and extensive evaluation metrics	Examples of interpretability should be provided for more than one timeframe
Li et al. (2020)	DRL hybrid with Adaboost ensemble that provides good performance	Discussion included ML evaluations that were not presented
Théate and Ernst (2020)	Extensive evaluation criteria with adequate consideration for trading cost	Details of backtesting not provided
Zhang et al. (2020d)	Includes tests across a vast amount of financial instruments and evaluation measures	Unclear on how or why cross-validation was combined with the backtesting approach that was employed to control overfitting.
Chakole and Kurhekar (2020)	Focus on market trends using extensive financial and ML evaluation metrics and incorporating transaction costs	Interpretability insights needed to provide context for the good performance
Wu et al. (2019)	Extensive evaluation metrics and well-defined backtesting strategy	Lacking conversation regarding interpretability
Hu et al. (2018b)	Uses chart representations of financial data as DL input, producing a good performance	Numerical representation of the same data is missing, precluding a balanced comparison. Furthermore, there is no discussion of model interpretability
Lei et al. (2020)	ResNet used to improve the effectiveness of moving average indicators in terms of financial and ML evaluation metrics	Minimal explanation for the model’s performance
Chen et al. (2018b)	Uses a significant amount of high-frequency trading data as input image in a pair trading setup using CNN	Minimal evaluation of returns and no evaluation results presented. Furthermore, there are no comparisons with the raw numerical data
Wu et al. (2020)	Uses a high-frequency trading technique to predict profitability on daily options trading using LSTM. Provides extensive details regarding the backtesting approach	No baseline comparison provided
Koshiyama et al. (2020)	Uses LSTM encoder-decoder to transfer trends across 58 different global markets with impressive results across multiple financial and ML evaluation metrics	No interpretation of the model’s operation or details of the featured transfer
Sun et al. (2019)	Predicts futures market movement using LSTM across multiple criteria, including simulated live trading. Additionally, multiple well backtested models are generated with different parameters and time windows	Lack of clarity regarding why the presented model’s accuracy is worse than chance. A baseline comparison with financial metrics could provide that clarity
Silva et al. (2020)	Clear presentation of the strategy, and evaluation across multiple financial and ML criteria	No insights or explanations regarding the output of the LSTM model employed
Wang et al. (2020)	Combines LSTM with market indicators in a novel manner with promising results	The presented evaluation is unclear, and no insights are offered regarding the model’s performance
Chalvatzis and Hristu-Varsakelis (2020)	Nine DL ML models are combined with LSTM in an ensemble; a well-formalized trading strategy, training, and testing conducted using a practical rolling windows approach and a complete set of evaluation criteria	Although the general discussion regarding evaluation is extensive, it does not provide insights into the model’s performance in relation to the input features
*Price prediction*		
Wang et al. (2019a)	Convolutional LSTM enables price prediction with improved performance while controlling for overfitting	Lack of discussion regarding explainability
Zhang et al. (2019)	Combines LSTM and CNN to capture spatial structure in LOB and features sufficient backtesting	Given the approach to backtesting, there is no indication of whether multiple models have been created or the same model is updated
Zhao et al. (2018)	Uses market charts as input for a CAE that serves as LSTM input	Approach to backtesting unclear due to the unusual data split across training, validation, and test sets. Furthermore, lacks sufficient baseline comparisons and offers no discussion regarding model explainability
Zhang et al. (2020b)	Combines LSTM with Autoencoder and CNN for improved predictive results across financial and ML metrics	Insufficient discussion regarding model explainability
Fang et al. (2019)	Regression model is combined with LSTM for better predictive performance	Concludes that the results are not stable for backtested data
Baek and Kim (2018)	Uses LSTM for data augmentation, specifically targeting controlling overfitting. Provides extensive results across ML, financial, and statistical criteria and discusses model performance	No justification for why the work only considered price data, rendering the provided model’s explanation less complete
Wang et al. (2018)	Uses one-dimensional CNN for price prediction, demonstrating better generalization than SVM and FFNN	Makes an argument against a buy-and-hold baseline; however evaluation results based on the argument would be sufficient evidence. More discussion regarding model explainability needed
Zhang et al. (2020c)	Provides good evaluation results for the use of an Autoencoder for feature reduction in an ensemble learning setup	Lack of clarity regarding the backtesting strategy and the data splits. Furthermore, no discussion provided regarding model explainability
*Portfolio management*		
Liang et al. (2018)	Early attempt at using DRL in the financial market featuring sufficient backtesting and evaluation of results	No discussion regarding model explainability. Furthermore, the paper concludes that the results are unfavorable
Park et al. (2020)	Uses Q Learning to derive trading strategies in a simulated feature space to gain experience beyond the available data. Impressive performance in comparison to the baseline	No discussion concerning insights into the model’s decisions
Guo et al. (2018)	Ensemble of portfolio management using the existing state-of-the-art strategy with DRL to provide a vast improvement on returns.	Lack of discussion regarding model explainability, a necessity for insights into the vastly improved performance
Wang and Wang (2019)	Uses ResNet to address overfitting problems when presented with noisy financial data. Sufficient backtesting results provided across statistical and financial metrics	No insights into model performance provided to help understand the data features contributing to the performance
*Market simulation*		
Maeda et al. (2020)	Uses DRL and LSTM to simulate market data, enabling the creation of theoretical market conditions with impressive results for returns compared to the baseline	Lack of discussion regarding model explainability
Buehler et al. (2020)	Provides a good overview of the theories involved in generative financial data modeling	Although there is an argument that no value is derived from using more data, it is worth investigating including a comparison with more kinds of real data based on multiple market scenarios
Raman and Leidner (2019)	Simulates up to a year of market data using only 6 weeks of real market data; simulated data is used with DRL for test trading decisions with sufficient baseline comparisons	No financial evaluation metrics for the simulated trades. Furthermore, performance implications of a longer time frame for input and simulated data would be useful
*Stock selection*		
Zhang et al. (2020a)	Combines LSTM with boosting ensembles to identify key market features and reduce overfitting	No comparisons with traditional feature reduction methods such as PCA and no discussion of model explainability
Amel-Zadeh et al. (2020)	Using only fundamental data, compares RNN and FFNN models with non-DL algorithms; the non-DL methods outperform the DL models	No information on the completeness of the input data, namely, lagged dates, and no insights into the model’s performance
Yang et al. (2019)	Compares CNN with LSTM, with features derived from profit indicators	No comparisons with other baseline strategies and no discussion of model explainability
*Risk management*		
Arimond et al. (2020)	Specifically targeted at using CNN and LSTM to estimate VaR with a focus on future research potentials	Fails to formally present the baseline evaluation results or provide an explanation or suggestions regarding potential model performance
*Hedging strategy*		
Ruf and Wang (2020)	Uses FFNN to predict a derived metric that it uses in a hedging strategy with promising results	No consideration of the state of model or discussion of insights from the model output

Table 12 presents the highlights of and problems with the studies reviewed, demonstrating that while all represent impressive work in different capacities, many insufficiently discuss model explainability, and none focus on the long-term investment horizon. Also, while these works mostly combine market and fundamental data, it is still difficult to include alternative data, such as news texts or Twitter data, which can enrich the modeling process. This is largely due to the unavailability of such data, especially for long historical time windows. The next section elaborates on these challenges. As this area of research continues to mature, we hope that more attention is paid to these issues and that researcher interests can influence the industry at large to make most of the cost-prohibitive data forms available for research purposes.

### Lessons learned

This section has focused on our research’s findings and methodology. While numerous studies have used stock market data for ML, readers will notice that very few works do the due diligence of backtesting as part of their experimentation. Of over 10,000 publications identified, only 35 papers meet this criterion. We have reviewed and summarized these contributions. These studies primarily focus on several specialization areas, and we have reviewed them on the basis of those specializations. Notably, the works considered were mostly published in the last 3 years and mostly based on market data from the US and China.

Upon analyzing the specific work items and methodologies in these papers, several simple patterns become obvious. For example, tasks depending heavily upon historical context—i.e., trading strategy and price prediction—commonly employ stateful architectures, such as DRL and LSTM, as the primary architecture to approach past market activities. Interestingly, although various of these problems have been formulated as online learning problems, the literature has not substantially established that connection. One of this work’s objectives has been to identify this blind spot such that, as the computer science research community matures in this area, it will be possible to leverage established practices to further improve the state-of-the-art.

The next section itemizes some of the interesting challenges identified during this survey and suggests future directions that can improve the field.

## Challenges and future directions

Previous sections have discussed what it means to conduct backtested DL research in the stock market context and summarized current research pursuing such a direction. Although there has been increasing focus on this area in recent years, numerous research challenges clearly remain. This section summarizes these challenges and provides suggested research directions.

### Challenges

#### Availability of historical market data

At the core of studies based on stock market analysis is the availability of consistently updated historical data. Unfortunately, such data is a premium product that is not readily available, especially at high levels of granularity (i.e., intraday and tick data). Paywalls often restrict access to such data, complicating its use for academic research, especially research without significant financial backing. Institutions such as Wharton Research Data Services (WRDS) (Wachowicz 2020) collaborate with academic institutions to provide access to some of these kinds of data. However, the degree of access is determined by the subscription level, which depends on the importance ascribed by the subscribing institution. Nonetheless, the data remain widely inaccessible to a larger pool of institutions, making the only options either inconsistent publicly available market data or paying the premium.

#### Access to supplementary data

Closely related to the previous issue is access to related data types, which can be used to improve performance on modeling tasks involving financial data. Examples include fundamental data (e.g., quarterly reports) and alternative data (e.g., news articles and tweets about the company of interest). It is important to differentiate these kinds of data because sources usually differ from those responsible for market data. Notably, Twitter recently announced API access for research purposes (Tornes and Truijillo 2021), which could help with this issue. However, there are many other kinds of potential supplementary data, and there remains some work to reach a state where such data is readily available. For example, it would be invaluable for news API services, such as webhose.io, to provide API access to supplementary news data for research purposes.

#### Long term investment horizon

Several studies reviewed consider a relatively short investment horizon, from a few days to a few months. Given a significant amount of investments in the stock market are associated with portfolios that span decades, such as retirement funds, buying and holding *growth investment* is attractive. Growth investment expects above-average returns for young public companies, with the expectation of significant future growth. For example, Shopify (SHOP) IPO-ed at $17 in May-2015; as of Feb-2020, a share was worth $${\sim }\$530$$, with the price ending the year at $${\sim }\$1100$$. This suggests that it was a growth investment at the early stage; identifying that character early would have produced larger than average returns. Such patterns could be discovered by using supplementary data as discussed. By modeling similar historical growth investments as part of an investment strategy, it might be possible to identify newer investments that can produce handsome returns for long-term investments.

#### Effect of capital gains tax

Several studies draw conclusions on strategy without considering trading costs or taxation. This is more pronounced for short-term investments, for which tax rates are high (10–37% in the US) compared to long-term investments (0–20%). Thus, to accurately represent returns, these costs must be considered; however, this is seldom done.

#### Financial ML/DL framework

Many popular ML and DL frameworks, including scikit-learn (Pedregosa et al. 2011), TensorFlow (Abadi et al. 2016), Keras (Chollet et al. 2015), PyTorch (Paszke et al. 2019), have improved the state-of-the-art. These frameworks are commonly used in both academic research and industrial research for production-level use cases. Although these frameworks appeared frequently in the studies reviewed, implementations generally corresponded to the respective financial considerations, that is, we observed no real attempts to extend existing frameworks using improvements based on these specialized works.

Stock market ML problems involve incrementally learning using time-series data. Although this represents an online learning problem, the similarity remains to be fully appreciated. For example, ideas commonly used for concept drift in online learning research (Lu et al. 2020) appear perfectly suited to regime switch in quantitative analysis research. Meanwhile, some ML frameworks that are dedicated to online learning research have the tools and consideration for concept drift and prequential evaluation built into their framework. These include scikit-multiflow (Montiel et al. 2018) and River (Montiel et al. 2020).

The absence of such frameworks for financial ML means that individual research teams must implement their ideas without attempting to integrate them into an open-source framework. Section 3.2.1 discusses protocols for ML research that involve proving results via backtesting. Having an accessible framework focused on DL research using financial data would enable the promotion of such ideas and allow research in this area to more closely conform to established industry practice. It would also enable researchers to provide specific implementations to improve the state-of-the-art, avoiding the current siloed approach that precludes real effort at cohesion.

### Future directions

The challenges identified in the previous section lead to several ideas for future research in this area:

- *Applicability in practice* This work’s focus has been on ensuring we attend to how previous works have been validated in practice. Industry applicability, trustworthiness, and usability (The Institute for Ethical AI & Machine Learning 2020; Gundersen et al. 2018) should be our core guiding forces as we expand computer science learnings and research into domain-specific applications such as the financial market. One approach is ensuring that we adhere to guiding protocols, such as backtesting, when conducting research experiments in the financial market context (Arnott et al. 2018). This aligns with pertinent AI research topics such as reproducibility and explainability (i.e., XAI).
- *Improvements in trust* Although significant attention has recently been focused on AI trustworthiness, there remains much work to be done. An important principle for building trust in AI is *explicability*, which entails creating explainable and accountable AI models (Thiebes et al. 2020). Ensuring that research is explicable further improves the chance of employing that research in real-world scenarios. Recall that Sect. 3.2.1 indicated that feature importance could provide explainable insights from input features, which, in turn, endow trust. There remains substantial work to be done on this matter, as the summaries provided in Table 12 evidence, especially the limited attention given to explainability. Another important point of tension for generating trust in AI is reproducibility. Among other considerations, publications must be easy to validate by external researchers. Notably, (Thiebes et al. 2020) provides a checklist including relevant statistical items and code and data availability. However, of the 35 papers reviewed, only seven (20%) provide the source code for their research. Ensuring that all published works include access to the source code and data would help increase trust, making industrial application more plausible.
- *Public availability of data* One means of improving trust in AI research is the availability of public data that researchers can use as a benchmark. Unfortunately, because this is relatively uncommon for financial market research, relevant fundamental (i.e., quarterly reports), alternative (i.e., news and social media), and granular/intraday market data are often behind paywalls. This means that even if most researchers were to publish their source code, they still might not be able to publish their data due to legal implications. While efforts made by corporate organizations such as Twitter is laudable (Tornes and Truijillo 2021), there remains work to be done by the industry and researchers to make relevant research data available for this purpose. An ideal set would be historical market data over a long period, with corresponding fundamental and alternative data sets. Although WRDS (Wachowicz 2020) is a good source of such for research purposes, research institutions must choose to subscribe and will provide varying levels of access based on financial commitment.
- *Focus on long-horizon* More emphasis should be made to apply DL market strategies to long-horizon investments targeted at growth investing. As previously mentioned, significantly more gains can be expected in the long-term investment horizon (i.e., > a year) by focusing on potential unicorns in their early stage. The consideration that one common investment portfolio type is retirement funds, which feature a relatively long time span, makes a compelling case for considering modeling techniques focused on long-term returns. However, a potential drawback is that this complicates evaluating annualized metrics, especially for longer-term objectives. A hybrid approach might be to mix a short-term strategy with a vision for the long term. Additionally, employing alternative data, such as news articles, about not only the company of interest but also competitors can enable longer-term horizons to be better forecast. Additionally, tracking either or both geopolitical and environmental events and their potential impacts to “learn from the past” represents an interesting future study direction.
- *Financial DL frameworks* Significant work has been done to apply ML to stock market research. However, unified frameworks remain uncommon, especially in DL research. Thus, a useful step would be to develop a financial DL toolbox for online learning using non-stationary financial data that are inherently volatile (Pesaranghader et al. 2016). Section 3.2.1 discussed the peculiarities of learning from non-stationary time-series data pertaining to the stock market. A unified financial DL toolbox improved by different research would help to foster innovation based on newer ideas.

## Conclusion

As DL becomes more common in financial research, it is apparent that attention is increasingly focused on ensuring that the research process conforms to procedures established in the financial domain. A recent example of this is the renewed attention on backtesting algorithms using historical data and domain-specific evaluation metrics. As neural processors become ubiquitous, traditionally compute-intensive algorithms become more attractive for online learning. Consequently, we expect to see DL increasingly applied to solving research problems using stock market data.

This survey involved reviewing backtested applications of DL in the stock market. The backtesting requirement indicates that the research has demonstrated some degree of due diligence, enabling consideration for real-world use. After demonstrating the nature of stock market data and common representations of these data, before and after some pre-processing for ML purposes to understanding the nuances of this type of data, we summarized DL architectures, focusing on those used in the literature reviewed. This enabled the quick establishment of points of reference for discussion of the architectures in the context of those studies.

While numerous studies have explored stock market applications of DL, we focused on those that demonstrate evidence of research methodology consistent with the domain and thus more likely to be considered by industry practitioners (Paleyes et al. 2020; The Institute for Ethical AI & Machine Learning 2020; Gundersen et al. 2018). In following this approach, it was hoped that this survey might serve as a basis for future research answering similar questions. To that end, we concluded the survey by identifying open challenges and suggesting future research directions. Our future work will aim to assist in addressing such challenges, especially through explorations of supplementary data and developing novel explainable financial DL frameworks.

## Appendix 1—Acronyms

**Table:**

AI	Artificial intelligence
ANN	Artificial neural networks
API	Application Programming Interface
CAE	Convoluted autoencoder
CAGR	Compound annual growth rate
CBOE	Chicago Board Options Exchange
CNN	Convolutional neural network
DBN	Deep belief networks
DL	Deep learning
DQN	Deep Q-Network
DRL	Deep reinforcement learning
EAIML	Ethical AI & machine learning
FFNN	Feed-forward neural networks
FIX	Financial information exchange
GRU	Gated recurrent unit
GTP	GPRS tunnelling protocol
HMM	Hidden markov model
IID	Independent and identically distributed
KLD	Kullback-Leibler divergence
LOB	Limit order book
LSTM	Long short-term memory
MACD	Moving average convergence/divergence
MAE	Mean absolute error
MAPE	Mean absolute percentage error
MDD	Maximum drawdown
MDP	Markov decision process
ML	Machine learning
MSE	Mean square error
NLP	Natural language processing
OHLC	Open-high-low-close
PCA	Principal Component Analysis
PReLU	Parametric ReLU
RBM	Restricted Boltzmann machine
RL	Reinforcement learning
RNN	Recurrent neural network
ReLU	Rectified linear unit
ResNet	Residual network
RoR	Rate of returns
SVM	Support vector machines
TQDN	Trading deep Q-network
VIX	Volatility index
VWAP	Volume-weighted average price
VaR	Value-at-risk
WRDS	Wharton research data services
XAI	Explainable AI

## Footnotes

1 Searched for “deep learning” AND “stock market” on Google Scholar

2 Query results as of November 5, 2020
